# Six New Phragmalin Limonoids from the Stems of *Chukrasia tabularis* A. Juss

**DOI:** 10.3390/molecules23113024

**Published:** 2018-11-20

**Authors:** Yan-Cui Wang, Fan-Dong Kong, Hao Wang, Wen-Li Mei, Shou-Bai Liu, You-Xing Zhao, Hao-Fu Dai

**Affiliations:** 1 Hainan Key Laboratory for Research and Development of Natural Products from Li Folk Medicine, Institute of Tropical Bioscience and Biotechnology, Chinese Academy of Tropical Agricultural Sciences, Haikou 571101, China; wangyancui.mail@163.com (Y.-C.W.); kongfandong@itbb.org.cn (F.-D.K.); wanghao@itbb.org.cn (H.W.); meiwenli@itbb.org.cn (W.-L.M.); zhaoyouxing@itbb.org.cn (Y.-X.Z.); 2 Institute of Tropical Agriculture and Forestry, Hainan University, Haikou 570228, China; zhiwu19831113@163.com

**Keywords:** *Chukrasia tabularis* A. Juss, Meliaceae, phragmalin limonoid, α-glucosidase inhibition activity, acetylcholinesterase inhibitory activity

## Abstract

Six new phragmalin limonoids, named moluccensin Z1 (**1**), moluccensin Z2 (**2**), carapanolide Y (**3**), tabulalin N (**4**), chukvelutilide A1 (**5**), and velutinasin J (**6**), as well as two known compounds, chukvelutilide A (**7**) and velutinasin D (**8**) were isolated from the stems of *Chukrasia tabularis* A. Juss. The structures of the new compounds **1**–**6** were confirmed by spectroscopic methods, including IR and HRESIMS, as well as 1D and 2D NMR, and by comparisons with the data of known analogues. All compounds were tested for α-glucosidase and acetylcholinesterase inhibitory activities. However, none of the compounds was active against α-glucosidase and acetylcholinesterase in vitro.

## 1. Introduction

*Chukrasia tabularis* A. Juss (Meliaceae) are distributed over the tropical areas of Asia, and its root bark has been used as a traditional medicine for dispelling wind and heat from the body in the Hainan province of China for a long time [1,2]. Previous chemical studies have reported a large number of structurally diverse limonoids from this genus [3], and some of them exhibited anti-inflammatory, antibacterial, insecticidal and cytotoxic activities [4,5,6,7,8]. Phragmalin limonoids such as normal phragmalins and their orthoesters, C(15)-acyl phragmalins, 16,19-dinorphragmalins, 13/14/18-cyclopropanyl phragmalin-type orthoesters, 16-dinorphragmalins, and C(15)-acyl 16-dinorphragmalins are the characteristic components of *Chukrasia* genus [9,10,11,12,13,14,15,16,17,18].

**Figure 1:**
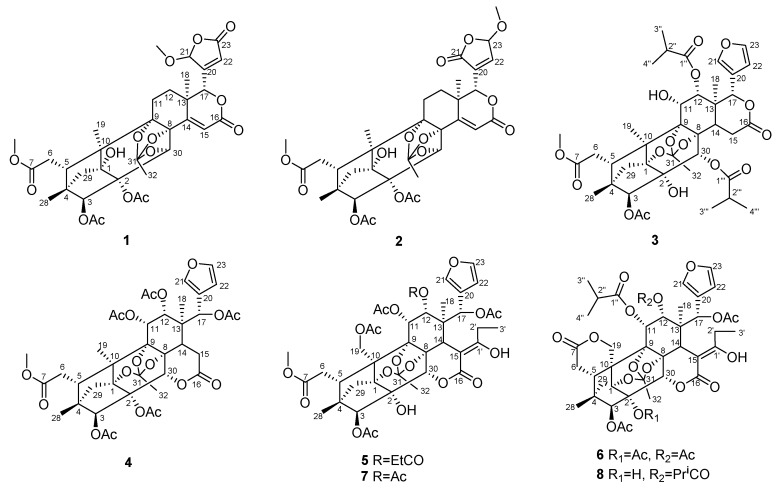
The chemical structures of compounds **1**–**8**.

In our previous study, some phragmalin limonoids such as chukbularisin B–E isolated from the big polar part of EtOAc-soluble extract of *C. tabularis* significantly inhibited the α-glucosidase [19]. As part of our investigation towards limonoids with novel structures, we continued to study on the small polar part of EtOAc-soluble extract of *Chukrasia tabularis* A. Juss, which afforded six new phragmalin limonoids, named moluccensin Z1 (**1**), moluccensin Z2 (**2**), carapanolide Y (**3**), tabulalin N (**4**), chukvelutilide A1 (**5**), and velutinasin J (**6**), together with two known compounds chukvelutilide A (**7**) and velutinasin D (**8**) (Figure 1). Compounds **1**–**8** were evaluated for the inhibitory effects on α-glucosidase and acetylcholinesterase. In this paper, the isolation, structural elucidation as well as the evaluations focused on the α-glucosidase and acetylcholinesterase inhibitory effects of eight limonoids from the stems of *C. tabularis* are described.

## 2. Results and Discussion

**Figure 2:**
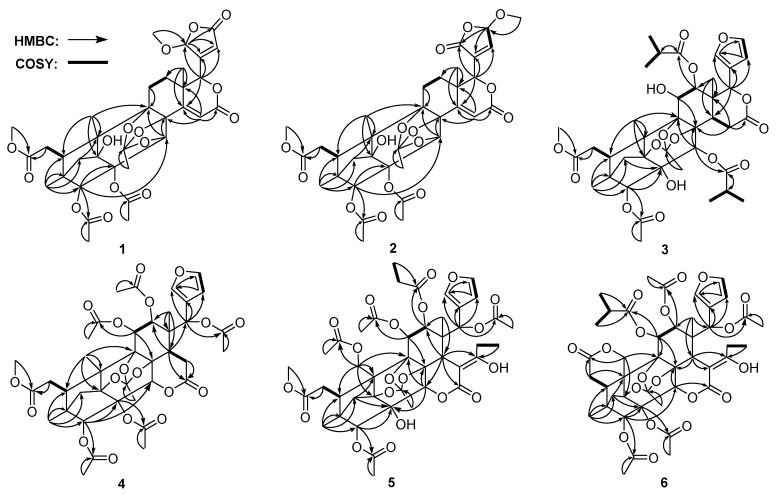
Key HMBC and ^1^H-^1^H COSY correlations for compounds **1**–**6**.

**Figure 3:**
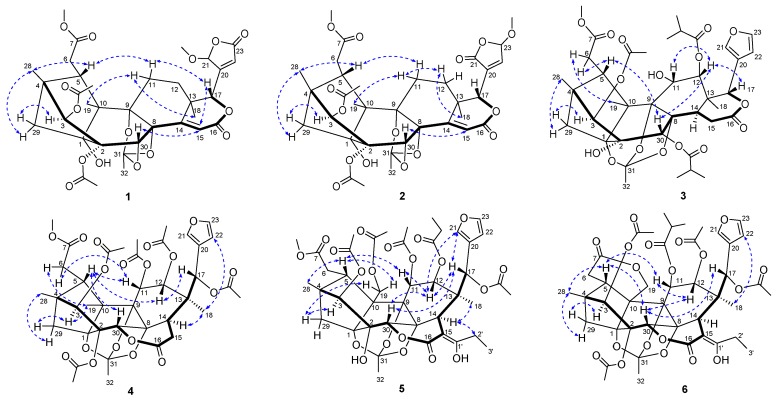
Key ROESY correlations for compounds **1**–**6**.

Compound **1**, a white amorphous powder, had a molecular formula of C_34_H_40_O_15_ as determined by the HRESIMS ion at *m*/*z* 711.2274 ([M + Na]^+^ calcd. 711.2259), corresponding to 15 degrees of unsaturation. The IR absorptions showed the presence of hydroxy group (3528 cm^−1^) and carbonyl group (1731 cm^−1^). The ^1^H-NMR (Table 1), ^13^C-NMR (Table 2) along with the HSQC data of **1** revealed the presence of two methoxy groups, two acetoxy groups, three ester carbons, four methyls, four methylenes, seven methines with four oxygenated, and ten quarternary carbons (two olefinic and four oxygenated). These data were similar to those of moluccensin Y [20], suggesting that compound **1** was also an 8,9,30-phragmalin *ortho* ester. The main differences between them were the presence of a lactone carbonyl (δ_C_ 169.0), a methoxy (δ_H_ 3.56; δ_C_ 57.3) and an acetal methine (δ_H_ 5.85; δ_C_ 103.8) signals and the absence of two olefinic methine signals in **1** compared to moluccensin Y. HMBC correlations between 21-OMe/C-21, H-21/C-20, H-21/C-22, H-21/C-23, H-17/C-20, H-17/C-21, and H-17/C-22 indicated that a β-furyl ring moiety located at C-17 in moluccensin Y was replaced by a 21-methoxy-20(22)-en-21,23-γ-lactone moiety in **1**. The remaining substructure was determined to be the same as moluccensin Y based on the 2D NMR data as shown in Figure 2. The nearly identical chemical shifts and *J*-values suggested that compound **1** and moluccensin Y shared the same relative configuration. This deduction was confirmed by ROESY correlations of H-3/H-29a, H-11a/Me-18, Me-19/H-11a, H-5/H-11b, H-11b/H-17, H-15/H-30, H-17/H-15, Me-28/H-5, and Me-28/H-29b (Figure 3). Therefore, the structure of **1**, named moluccensin Z1, was established as shown.

Compound **2** was obtained as a white amorphous powder. The molecular formula C_34_H_40_O_15_ was determined by the pseudomolecular ion peak at *m*/*z* 711.2260 ([M + Na]^+^ calcd. 711.2259) in the HRESIMS, indicating 15 degrees of unsaturation. The IR spectrum of **2** displayed absorptions for hydroxy group at 3545 cm^−1^ and carbonyl group at 1734 cm^−1^. The NMR data of **2** (Table 1 and Table 2) showed great similarity to those of moluccensin Z1 (**1**), except for the replacement of the 21-methoxy-20(22)-en-21,23-γ-lactone moiety located at C-17 in **1** by a 23-methoxy-20(22)-en-21,23-γ-lactone moiety in **2**. This deduction was further confirmed by the HMBC correlation between H-17/C-20, H-17/C-21, H-17/C-22, H-23/C-20, H-23/C-21, 23-OMe/C-23, and ^1^H-^1^H COSY correlation of H-22/H-23 (Figure 2). The relative configuration of **2** was assigned to be the same as that of **1** based on the explanation of ROESY correlations (Figure 3). Thus, the structure of **2**, named moluccensin Z2, was elucidated as shown.

Compound **3**, a white amorphous powder, had the molecular formula of C_39_H_50_O_16_ as determined by the HRESIMS ion at *m*/*z* 797.3017 ([M + Na]^+^ calcd. 797.2991), which indicated 15 degrees of unsaturation. The IR absorption bands at 3457 cm^−1^ and 1736 cm^−1^ suggested the presence of hydroxy and carbonyl groups. Analysis of the ^1^H- and ^13^C-NMR data of **3** (Table 1 and Table 2) revealed that it was similar to those of carapanolide M [21], except for the replacements of the 12-*O*-acetyl group and 30-*O*-propionyl group in carapanolide M by the 12-*O*-isobutyryl group and 30-*O*-isobutyryl group in **3**, which was confirmed by COSY correlations of H-4″/H-2″/H-3″ and H-4‴/H-2‴/H-3‴ in combination with HMBC correlations of H-2″/C-1″ and H-2‴/C-1‴ (Figure 2). The relative configuration of **3** was established to be the same as that of carapanolide M by the ROESY spectrum (Figure 3). Therefore, the structure of **3** was elucidated and it was named carapanolide Y.

Compound **4** was isolated as a white amorphous powder. The molecular formula C_39_H_46_O_18_ was determined by the HRESIMS ion at *m*/*z* 825.2588 ([M + Na]^+^ calcd. 825.2576), which indicated 17 degrees of unsaturation. The IR spectrum of **4** exhibited absorption for carbonyl groups at 1750 cm^−1^. The ^1^H- and ^13^C-NMR data of **4** (Table 2 and Table 3) were similar to those of tabulalin C [22]. Compared with tabulalin C, **4** had three acetoxy groups, which replaced 2-OH, 3-OH and H-11 in tabulalin C, respectively, and lacked an acetoxy group at C-19. The methyl at C-19 was confirmed by the HMBC correlations between H-19/C-10, H-19/C-5 and H-19/C-9. The acetoxy groups at C-2, C-3 and C-11 were revealed by the HMBC correlations from H-2, H-3 and H-11 to the corresponding carbonyl of the acetoxy group (Figure 2). The relative configuration of **4** was established to be the same as these of tabulalin C based on the explanation of ROESY correlations (Figure 3). Thus, the structure of **4** was assigned as depicted and it was named tabulalin N.

Compound **5**, a white amorphous powder, had the molecular formula of C_43_H_52_O_20_ as determined by the HRESIMS ion at *m*/*z* 911.2932 ([M + Na]^+^ calcd. 911.2944), which indicated 18 degrees of unsaturation. The IR absorption bands at 3451 cm^−1^ and 1743 cm^−1^ suggested the presence of hydroxy and carbonyl groups. The ^1^H- and ^13^C-NMR data of **5** (Table 2 and Table 3) showed great similarity to those of chukvelutilide A [11]. The only difference was the replacement of the 12-*O*-acetyl group in chukvelutilide A by the 12-*O*-propionyl group in **5**, which was further confirmed by HMBC and ^1^H-^1^H COSY correlations as depicted in Figure 2. The relative configuration of **5** was established to be the same as that of chukvelutilide A by the ROESY spectrum (Figure 3). Therefore, the structure of **5** was elucidated and it was named chukvelutilide.

Compound **6** was obtained as a white amorphous powder. The molecular formula C_43_H_50_O_19_ was determined by the pseudomolecular ion peak at *m*/*z* 893.2822 ([M + Na]^+^ calcd. 893.2839) in the HRESIMS, indicating 19 degrees of unsaturation. The IR spectrum of **6** displayed absorptions for hydroxy group at 3481 cm^−1^ and carbonyl groups at 1748 cm^−1^. The ^1^H- and ^13^C-NMR data of **6** (Table 2 and Table 3) were similar to those of velutinasin D [23], except for the replacements of the 12-*O*-isobutyryl group and 2-OH in velutinasin D by the 12-*O*-acetyl and 2-*O*-acetyl in **6**. The acetoxy at C-12 (δ_C_ 69.6) was revealed by the HMBC correlations from H-12 (δ_H_ 4.72) to the corresponding carbonyl of the acetoxy group. Similarly, the acetoxy at C-2 was confirmed by the HMBC correlations (Figure 2). The relative configuration of **6** was established to be the same as that of velutinasin D by the ROESY spectrum (Figure 3). Thus, the structure of **6**, named velutinasin J, was elucidated as shown.

Two known compounds were identified as chukvelutilide A (**7**) [11] and velutinasin D (**8**) [23], respectively, by interpreting their NMR data and making comparisons with literature values. More details about the original spectra of NMR, IR and HRESIMS data for the new compounds **1**–**6** see Figures S1–S48 of the supplementary materials.

All the compounds were tested for the α-glucosidase and acetylcholinesterase inhibition activities according to the method of Li [24] and Xiang [25]. There was no obvious inhibition effect on α-glucosidase and acetylcholinesterase. Previous research showed that the EtOAc-soluble extract of *C. tabularis* and some phragmalin limonoids which were isolated from it had significant α-glucosidase inhibitory activity [19]. Compare the chemical structures between the previously isolated limonoids with significant α-glucosidase inhibitory activities and the newly isolated compounds, a quinary lactone ring instead of a β-furyl ring located at C-17 in compound **1** and **2**, D-rings were opened and an acetoxy group was connected to C-17 in compounds **4**–**8**. These differences of chemical structures might be the reason for missing the α-glucosidase inhibitory activity of the newly isolated compounds, and were consistent with the result of our previous study [19].

## 3. Materials and Methods

### 3.1. General Procedures

The NMR spectra were recorded with a Bruker AV III spectrometer (Bruker, Bremen, Germany) using TMS as an internal standard. Optical rotations were measured on an MCP 5100 polarimeter (Anton Paar, Graz, Austria). The infrared spectra were recorded with a Nicolet 380 FT-IR spectrometer (Thermo, Pittsburgh, PA, USA). UV spectra were recorded on a Shimadzu UV2550 spectrophotometer (Shimadzu, Kyoto, Japan). The mass spectrometric (HRESIMS) data were acquired using an API QSTAR Pulsar mass spectrometer (Bruker, Bremen, Germany). Melting points were obtained with an apparatus of Beijing Taike X-5 (Beijing Taike Instrument Co. Ltd., Beijing, China). MCI gel CHP-20P (75–150 µm; Mitsubishi Chemical Industries Co. Ltd., Tokyo, Japan), silica gel (60–80 and 200–300 mesh; Qingdao Haiyang Chemical Co. Ltd., Qingdao, China), Rp-C_18_ (20–45 µm; Fuji Silysia Chemical Ltd., Durham, NC, USA) and Sephadex LH-20 (40–70 µm; Merck, Darmstadt, Germany) were used for column chromatography. Preparative HPLC was performed using an Agilent Technologies 1260 Infinity equipped with a YMC-packed Rp-C_18_ column (5 μm, 250 mm × 10 mm, 4 mL/min) and an Agilent DAD G1315D detector. The solvents used to the extraction or isolation of the columns (MCI gel, Silica gel, Sephadex LH-20 and Rp-C_18_ columns), such as ethyl acetate, methanol, chloroform and methanol, were of analytical pure (Concord Technology Co. Ltd., Tianjin, China). The solvents used to the preparative HPLC, such as methanol and acetonitrile, were of chromatographic grade (Concord Technology Co. Ltd., Tianjin, China).

### 3.2. Plant Material

The stems of *C. tabularis* were collected from Haikou, Hainan Province, P.R. China, in July 2014, and identified by Dr. Jun Wang. A voucher sample (No. 20140726) was deposited at the Institute of Tropical Bioscience and Biotechnology, Chinese Academy of Tropical Agriculture Science.

### 3.3. Extraction and Isolation

The dried stems of *C. tabularis* (110.0 kg) were pulverized and extracted three times with 95% ethanol (314 L) at room temperature. The extract was concentrated under reduced pressure to afford a crude extract (13.7 kg), followed by suspension in H_2_O and extraction with petroleum ether, EtOAc, and n-BuOH successively. Then, the extract solutions were evaporated to dryness under reduced pressure separately to get the petroleum ether extract (30.0 g), EtOAc extract (1700.0 g) and n-BuOH extract (800.0 g). The EtOAc extract (1700.0 g) was chromatographed on silica gel eluted with a petroleum ether-EtOAc system (20:1 to 0:1, *v*/*v*) to yield 18 fractions. Fr.15 (220.0 g) was further chromatographed on silica gel eluted with CHCl_3_-MeOH (50:1, *v*/*v*) to yield one fraction (90.0g), followed by MCI gel eluting with MeOH-H_2_O (from 4:6 to 1:0) to yield Fr.15-1–Fr.15-12. Fr.15-9 (30.0 g) was chromatographed on Sephadex LH-20 gel with MeOH to yield Fr.15-9-1–Fr.15-9-3. Fr.15-9-2 (5.0 g) was chromatographed on a reversed-phase C_18_ silica gel column eluted with acetonitrile–H_2_O (from 4:6 to 6:4) to provide eleven fractions (Fr.15-9-2-1–Fr.15-9-2-11). Fr.15-9-2-1 (140 mg) was separated by preparative HPLC [mobile phase: Acetonitrile/H_2_O (35:65, *v*/*v*); flow rate: 4 mL·min^−1^; UV detection at 214 nm] to obtain compound **1** (4.0 mg, t_R_ = 24.801 min) and compound **2** (7.4 mg, t_R_ = 26.522 min), respectively. Fr.15-9-2-4 (400 mg) was chromatographed on a reversed-phase C_18_ column eluted with MeOH-H_2_O (from 5:6 to 7:3) to give six subfractions (Fr.15-9-2-4-1–Fr.15-9-2-4-6). Fr.15-9-2-4-6 (8 mg) was separated by preparative HPLC [mobile phase: acetonitrile/H_2_O (57:43, *v*/*v*); flow rate: 4 mL·min^−1^; UV detection at 214 nm] to obtain compound **3** (2.0 mg, t_R_ = 12.014 min). Fr.15-9-2-3 (31 mg) was separated by preparative HPLC [mobile phase: MeOH/H_2_O (55:45, *v*/*v*); flow rate: 4 mL·min^−1^; UV detection at 214 nm] to obtain compound **4** (7.0 mg, t_R_ = 33.912 min). Fr.15-9-2-8 (170 mg) was chromatographed on a reversed-phase C_18_ column eluted with MeOH-H_2_O (from 6:4 to 7:3) to give six subfractions (Fr.15-9-2-8-1–Fr.15-9-2-8-6). Fr.15-9-2-8-5 (16 mg) was further separated by preparative HPLC [mobile phase: acetonitrile/H_2_O (55:45, *v*/*v*); flow rate: 4 mL·min^−1^; UV detection at 273 nm] to obtain compound **5** (4.0 mg, t_R_ = 21.674 min). Fr.15-9-2-11 (300 mg) was chromatographed on silica gel eluted with a petroleum ether–CHCl_3_–isopropanol system (100:40:1 to 40:40:1, *v*/*v*/*v*) to yield three subfractions (Fr.15-9-2-11-1–Fr.15-9-2-11-3). Fr.15-9-2-11-2 (78 mg) was further separated by preparative HPLC [mobile phase: MeOH/H_2_O (72:28, *v*/*v*); flow rate: 4 mL·min^−1^; UV detection at 273 nm] to obtain compound **6** (14.0 mg, t_R_ = 10.340 min). Fr.15-9-2-11-3 (58 mg) was separated by preparative HPLC [mobile phase: MeOH/H_2_O (73:27, *v*/*v*); flow rate: 4 mL·min^−1^; UV detection at 273 nm] to obtain compound **8** (12.0 mg, t_R_ = 14.010 min). Fr.15-9-2-5 (770 mg) was first subjected to a reversed-phase C_18_ column eluted with MeOH-H_2_O (from 5:5 to 7:3) to give eight subfractions (Fr.15-9-2-5-1–Fr.15-9-2-5-8). Fr.15-9-2-5-6 (88 mg) was separated by preparative HPLC [mobile phase: MeOH/H_2_O (65:35, *v*/*v*); flow rate: 4 mL·min^−1^; UV detection at 273 nm] to obtain compound **7** (20.0 mg, t_R_ = 13.467 min).

*Moluccensin Z1* (**1**): White amorphous powder; m.p. 153–157 °C; ${\left[\alpha \right]}_{D}^{25}$ = +61.3° (*c* 0.08, MeOH); IR (KBr) ν_max_ 3528, 2924, 1731, 1457, 1372, 1260, 1094, 801, 736 cm^−1^; ^1^H- and ^13^C-NMR data see Table 1 and Table 2; positive-mode HRESIMS *m*/*z* 711.2274 [M + Na]^+^ (calcd. for C_34_H_40_O_15_Na, 711.2259).

*Moluccensin Z2* (**2**): White amorphous powder; m.p. 155–157 °C; ${\left[\alpha \right]}_{D}^{25}$ = +85.0° (*c* 0.08, MeOH); IR (KBr) ν_max_ 3545, 2925, 1734, 1458, 1371, 1260, 1027, 801, 737 cm^−1^; ^1^H- and ^13^C-NMR data see Table 1 and Table 2; positive-mode HRESIMS *m*/*z* 711.2260 [M + Na]^+^ (calcd. for C_34_H_40_O_15_Na, 711.2259).

*Carapanolide Y* (**3**): White amorphous powder; m.p. 120–123 °C; ${\left[\alpha \right]}_{D}^{25}$ = −36.3° (*c* 0.08, MeOH); λ_max_ (log ε) 306 (2.61) nm; IR (KBr) ν_max_ 3557, 2924, 1736, 1467, 1372, 1260, 1026, 801 cm^−1^; ^1^H- and ^13^C-NMR data see Table 1 and Table 2; positive-mode HRESIMS *m*/*z* 797.3017 [M + Na]^+^ (calcd. for C_39_H_50_O_16_Na, 797.2991).

*Tabulalin N* (**4**): White amorphous powder; m.p. 162–164 °C; ${\left[\alpha \right]}_{D}^{25}$ = −20.0° (*c* 0.20, MeOH); λ_max_ (log ε) 264 (2.92) nm; IR (KBr) ν_max_ 2923, 1750, 1372, 1217, 1026, 801 cm^−1^; ^1^H- and ^13^C-NMR data see Table 2 and Table 3; positive-mode HRESIMS *m*/*z* 825.2588 [M + Na]^+^ (calcd. for C_39_H_46_O_18_Na, 825.2576).

*Chukvelutilide* A1(**5**): White amorphous powder; m.p. 139–142 °C; ${\left[\alpha \right]}_{D}^{25}$ = −21.3° (*c* 0.08, MeOH); λ_max_ (log ε) 203 (3.86), 237 (3.32), 268 (3.57) nm; IR (KBr) ν_max_ 3451, 2918, 1743, 1373, 1218, 1026, 801 cm^−1^; ^1^H- and ^13^C-NMR data see Table 2 and Table 3; positive-mode HRESIMS *m*/*z* 911.2932 [M + Na]^+^ (calcd. for C_43_H_52_O_20_Na, 911.2944).

*Velutinasin J* (**6**): White amorphous powder; m.p. 169–171 °C; ${\left[\alpha \right]}_{D}^{25}$ = −3.5° (*c* 0.20, MeOH); λ_max_ (log ε) 234 (3.43), 269 (3.80) nm; IR (KBr) ν_max_ 3481, 2923, 1748, 1604, 1371, 1224, 1027, 800 cm^−1^; ^1^H- and ^13^C-NMR data see Table 2 and Table 3; positive-mode HRESIMS *m*/*z* 893.2822 [M + Na]^+^ (calcd. for C_43_H_50_O_19_Na, 893.2839).

## Figures and Tables

**Table 1:** ^1^H-NMR (500 MHz) data of compounds **1**–**3** (CDCl_3_, δ_H_ in ppm, *J* in Hz).

Proton	1	2	3
**3**	5.23 (s)	5.24 (s)	4.72 (s)
**5**	2.23 (m)	2.30 (m)	3.04 (d, 10.1)
**6a**	2.33 (br d, 10.0)	2.46 (br d, 15.6)	2.39 (dd, 16.9, 10.7)
**6b**	2.32 (br d, 10.0)	2.33 (br d, 15.6)	2.54 (br d, 16.9)
**11a**	2.23 (m)	2.17 (m)	
**11b**	2.01 (m)	1.90 (m)	4.29 (d, 2.3)
**12a**	1.45 (m)	1.17 (m)	
**12b**	1.62 (m)	1.26 (m)	4.54 (d, 2.3)
**14**			2.76 (overlap)
**15a**			2.76 (overlap)
**15b**	6.59 (s)	6.61 (s)	3.23 (dd, 17.4, 1.5)
**17**	5.67 (s)	5.55 (s)	5.66 (s)
**18**	1.41 (3H, s)	1.33 (3H, s)	1.42 (3H, s)
**19**	1.29 (3H, s)	1.26 (3H, s)	1.13 (3H, s)
**21**	5.85 (s)		7.44 (s)
**22**	6.25 (s)	7.37 (t, 1.5)	6.42 (dd, 1.9, 0.8)
**23**		5.80 (t, 1.5)	7.36 (t, 1.9)
**28**	0.72 (3H, s)	0.73 (3H, s)	0.90 (3H, s)
**29a**	1.72 (m)	1.70 (m)	1.78 (d, 11.5)
**29b**	1.94 (d, 11.6)	1.93 (d, 11.5)	1.89 (d, 11.5)
**30**	5.34 (s)	5.36 (s)	6.12 (s)
**32**	1.70 (3H, s)	1.69 (3H, s)	1.76 (3H, s)
**7-OMe**	3.64 (3H, s)	3.62 (3H, s)	3.73 (3H, s)
**21/23-OMe**	3.56 (3H, s)	3.60 (3H, s)	
**2-OAc**	2.16 (3H, s)	2.16 (3H, s)	
**3-OAc**	2.08 (3H, s)	2.11 (3H, s)	2.28 (3H, s)
**12-OCOCHMe_2_**			2.59 (m), 1.19 (3H, d, 7.2), 1.09 (3H, d, 6.8)
**30-OCOCHMe_2_**			2.17 (m), 0.95 (3H, d, 7.0), 0.83 (3H, d, 7.0)

**Table 2:** ^13^C-NMR (125 MHz) data of compounds **1**–**6** (CDCl_3_, δ_C_ in ppm).

Carbon	1	2	3	4	5	6
**1**	84.4	84.3	85.3	84.9	84.5	84.9
**2**	83.9	84.1	80.0	83.2	77.0	82.9
**3**	85.2	85.4	82.9	80.2	83.0	81.0
**4**	44.8	44.6	45.4	46.6	45.9	46.8
**5**	39.8	38.8	35.6	35.7	36.9	34.6
**6**	33.8	33.3	33.6	33.1	32.2	30.9
**7**	173.4	172.6	172.3	173.1	173.2	171.8
**8**	84.1	84.1	86.0	78.3	80.5	79.2
**9**	86.2	87.0	86.2	83.2	83.0	82.6
**10**	48.1	47.7	45.6	46.3	47.6	45.7
**11**	26.5	26.5	69.8	68.8	69.5	69.3
**12**	29.2	29.2	71.4	70.6	70.3	69.6
**13**	38.6	38.3	38.9	42.6	44.9	44.8
**14**	153.9	154.1	42.5	42.5	43.8	44.2
**15**	122.3	122.1	26.9	27.9	92.2	91.9
**16**	162.3	163.1	169.9	167.7	170.1	169.8
**17**	81.0	78.7	77.0	71.0	70.3	69.7
**18**	21.0	19.5	15.7	18.0	18.1	18.0
**19**	15.7	15.6	16.5	16.4	66.1	68.0
**20**	159.5	133.6	121.3	121.9	122.2	121.9
**21**	103.8	168.6	140.8	142.2	141.4	141.1
**22**	124.3	149.0	110.3	109.9	110.0	110.1
**23**	169.0	102.7	143.0	142.8	142.7	143.4
**28**	14.6	14.6	14.3	14.7	14.5	14.4
**29**	39.7	40.0	39.8	40.6	39.7	39.3
**30**	74.3	74.0	70.1	74.1	73.9	73.6
**31**	120.1	120.1	119.4	119.2	120.0	119.7
**32**	16.7	16.8	21.3	21.0	21.0	20.8
**1′**					180.1	180.8
**2′**					25.9	25.8
**3′**					11.3	11.3
**7-OMe**	52.4	52.4	51.9	52.1	52.1	
**21/23-OMe**	57.3	57.9				
**2-OAc**	170.7, 22.0	170.7, 22.0		169.5, 21.9		169.6, 21.8
**3-OAc**	169.3, 21.8	169.5, 21.8	171.1, 21.6	170.3, 21.0	170.5, 21.0	169.5, 21.3
**11-OAc/** **11-OCOCHMe_2_**				169.5, 20.8	168.9, 21.0	175.4, 34.2, 18.6, 19.5
**12-OAc/** **12-OCOCHMe_2_/** **12-OCOCHCH_2_Me**			175.3, 34.9, 19.5, 18.6	169.5, 19.8	172.3, 26.6, 8.5	169.8, 20.0
**17-OAc**				168.9, 21.3	169.0, 20.9	168.9, 20.7
**19-OAc**					171.1, 21.2	
**30-OCOCHMe_2_**			175.0, 33.8, 18.1, 18.1			

**Table 3:** ^1^H-NMR (500 MHz) data of compounds **4**–**6** (CDCl_3_, δ_H_ in ppm, *J* in Hz).

Proton	4	5	6
**3**	5.45 (s)	4.87 (s)	5.48 (s)
**5**	2.98 (d, 10.6)	3.21 (d, 10.1)	2.63 (m)
**6a**	2.44 (dd, 17.0, 10.6)	2.41 (m)	2.63 (m)
**6b**	2.78 (d, 17.0)	3.23 (d, 16.9)	2.99 (dd, 18.1, 5.4)
**11**	5.54 (d, 2.6)	6.42 (d, 2.3)	5.46 (d, 2.3)
**12**	4.53 (d, 2.6)	4.58 (d, 2.3)	4.72 (d, 2.3)
**14**	2.63 (d, 7.9)	3.37 (s)	3.23 (s)
**15a**	2.91 (dd, 18.6, 7.9)		
**15b**	3.11 (d, 18.6)		
**17**	5.88 (s)	5.90 (s)	5.80 (s)
**18**	1.48 (3H, s)	1.44 (3H, s)	1.59 (3H, s)
**19a**	1.22 (3H, s)	4.26 (d, 11.7)	4.68 (d, 14.2)
**19b**		4.54 (d, 11.7)	4.76 (d, 14.2)
**21**	7.64 (s)	7.60 (s)	7.34 (s)
**22**	6.42 (s)	6.40 (s)	6.31 (d, 1.4)
**23**	7.28 (t-like, 1.7)	7.26 (t-like, 1.6)	7.34 (t-like, 1.4)
**28**	0.96 (3H, s)	0.99 (3H, s)	1.11 (3H, s)
**29a**	1.82 (d, 11.2)	1.92 (d, 11.5)	1.84 (d, 11.5)
**29b**	2.01 (d, 11.2)	1.87 (d, 11.5)	2.44 (d, 11.5)
**30**	5.74 (s)	5.51 (s)	5.40 (s)
**32**	1.64 (3H, s)	1.64 (3H, s)	1.60 (3H, s)
**2′**		2.41, 2.58 (2H, m)	2.44, 2.63 (2H, m)
**3′**		1.26 (3H, t, 6.5)	1.24 (3H, t, 6.5)
**7-OMe**	3.71 (3H, s)	3.72 (3H, s)	
**2-OAc**	2.08 (3H, s)		2.10 (3H, s)
**3-OAc**	2.36 (3H, s)	2.36 (3H, s)	2.34 (3H, s)
**11-OAc/11-OCOCHMe_2_**	2.10 (3H, s)	2.11 (3H, s)	2.63 (m) 1.21 (3H, d, 6.9) 1.25 (3H, d, 7.2)
**12-OAc/12-OCOCHCH_2_Me**	1.57 (3H, s)	1.89, 1.76 (2H, m) 0.84 (3H, t, 7.5)	1.68 (3H, s)
**17-OAc**	2.04 (3H, s)	1.97 (3H, s)	1.92 (3H, s)
**19-OAc**		2.07 (3H, s)	
**1′-OH**		13.56 (s)	13.69 (s)

## Supplementary Materials

The following are available online. 1D- and 2D-NMR, IR, as well as HRESIMS spectra of Compounds **1**–**6**.
