# Supplementary material for: Six New Phragmalin Limonoids from the Stems of Chukrasia tabularis A. Juss
Source: Molecules. 2018 Nov 20;23(11):3024. doi: 10.3390/molecules23113024 (PMC6278448; doi:10.3390/molecules23113024)
Supplement: Supplementary file 1 [file molecules-23-03024-s001.pdf]

Supplementary Materials

Six New Phragmalin Limonoids from the Stems of *Chukrasia tabularis* A. Juss

Yan-Cui Wang [1], Fan-Dong Kong [1], Hao Wang [1], Wen-Li Mei [1], Shou-Bai Liu [2], You-Xing Zhao [1] and Hao-Fu Dai [1,*]

[1] Hainan Key laboratory for research and development of natural products from Li folk medicine, Institute of Tropical Bioscience and Biotechnology, Chinese Academy of Tropical Agricultural Sciences, Haikou 571101, China; wangyancui.mail@163.com (Y.-C.W.); kongfandong@itbb.org.cn (F.-D.K.); wanghao@itbb.org.cn (H.W.); meiwenli@itbb.org.cn (W.-L.M.); zhaoyouxing@itbb.org.cn (Y.-X.Z.)

[2] Institute of Tropical Agriculture and Forestry, Hainan University, Haikou 570228, China; zhiwu19831113@163.com

* Correspondence: daihaofu@itbb.org.cn; +86-898-6696–1869

Contents

Figure S1. HR-ESI-MS spectrum of **1** S4

Figure S2. ^{1}H-NMR spectrum ($CDCl_3$, 500 MHz) of **1** S4

Figure S3. ^{13}C-NMR spectrum ($CDCl_3$, 125 MHz) of **1** S5

Figure S4. ^{1}H-^{1}H COSY spectrum of **1** S5

Figure S5. ROESY spectrum of **1** S6

Figure S6. HMBC spectrum of **1** S6

Figure S7. HSQC spectrum of **1** S7

Figure S8. IR spectrum of **1** S7

Figure S9. HR-ESI-MS spectrum of **2** S8

Figure S10. ^{1}H-NMR spectrum ($CDCl_3$, 500 MHz) of **2** S8

Figure S11. ^{13}C-NMR spectrum ($CDCl_3$, 125 MHz) of **2** S9

Figure S12. ^{1}H-^{1}H COSY spectrum of **2**. S9

Figure S13. ROESY spectrum of **2** S10

Figure S14. HMBC spectrum of **2** S10

Figure S15. HSQC spectrum of **2** S11

Figure S16. IR spectrum of **2** S11

Figure S17. HR-ESI-MS spectrum of **3**........ S12

Figure S18. ^{1}H-NMR spectrum ($CDCl_3$, 500 MHz) of **3**........ S12

Figure S19. ^{13}C-NMR spectrum ($CDCl_3$, 125 MHz) of **3**........ S13

Figure S20. ^{1}H-^{1}H COSY spectrum of **3**........ S13

Figure S21. ROESY spectrum of **3**........ S14

Figure S22. HMBC spectrum of **3** S14

Figure S23. HSQC spectrum of **3**........ S15

Figure S24. IR spectrum of **3**........ S15

Figure S25. HR-ESI-MS spectrum of **4**........ S16

Figure S26. ^{1}H-NMR spectrum ($CDCl_3$, 500 MHz) of **4**........ S16

Figure S27. ^{13}C-NMR spectrum ($CDCl_3$, 125 MHz) of **4**........ S17

Figure S28. ^{1}H-^{1}H COSY spectrum of **4**. S17

Figure S29. ROESY spectrum of **4**........ S18

Figure S30. HMBC spectrum of **4** S18

Figure S31. HSQC spectrum of **4**........ S19

Figure S32. IR spectrum of **4**........ S19

Figure S33. HR-ESI-MS spectrum of **5**........ S20

Figure S34. ^{1}H-NMR spectrum ($CDCl_3$, 500 MHz) of **5**........ S20

Figure S35. ^{13}C-NMR spectrum ($CDCl_3$, 125 MHz) of **5**........ S21

Figure S36. ^{1}H-^{1}H COSY spectrum of **5**. S21

Figure S37. ROESY spectrum of **5**........ S22

Figure S38. HMBC spectrum of **5** S22

Figure S39. HSQC spectrum of **5**........ S23

Figure S40. IR spectrum of **5**........ S23

Figure S41. HR-ESI-MS spectrum of **6**........ S24

Figure S42. ^{1}H-NMR spectrum ($CDCl_3$, 500 MHz) of **6**.......... S24

Figure S43. ^{13}C-NMR spectrum ($CDCl_3$, 125 MHz) of **6**.......... S25

Figure S44. ^{1}H-^{1}H COSY spectrum of **6**. S25

Figure S45. ROESY spectrum of **6**.......... S26

Figure S46. HMBC spectrum of **6** S26

Figure S47. HSQC spectrum of **6**.......... S27

Figure S48. IR spectrum of **6**.......... S27

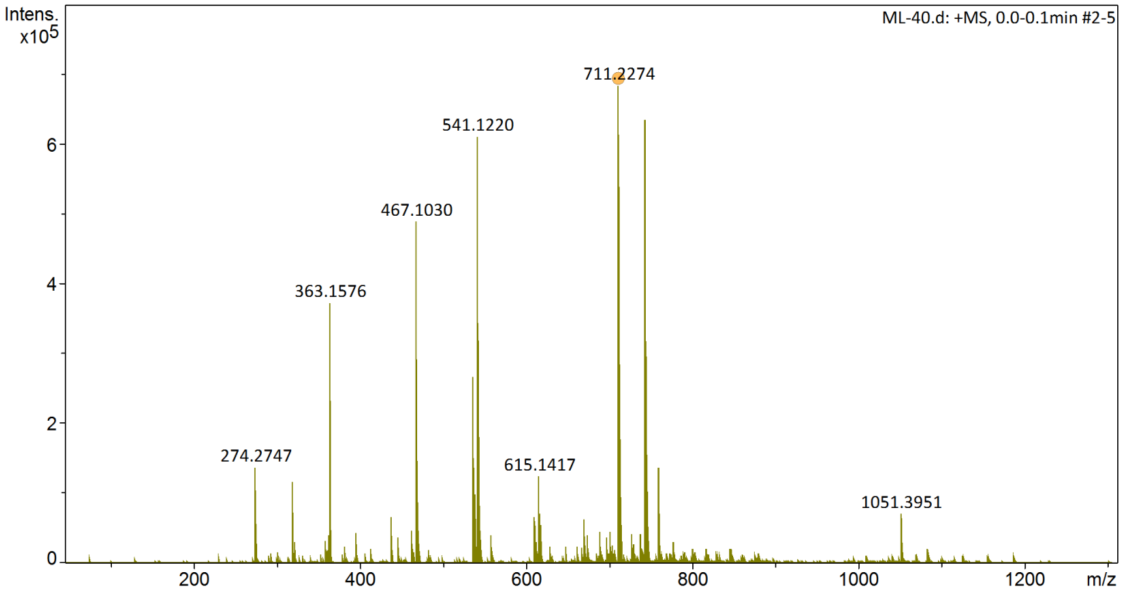

Sum Formula	Ion Formula	Meas. m/z	m/z	err [mDa]	err [ppm]
C34H40O15	C34H40NaO15	711.2274	711.2259	-1.4	-2.0

Figure S1. HR-ESI-MS spectrum of **1**

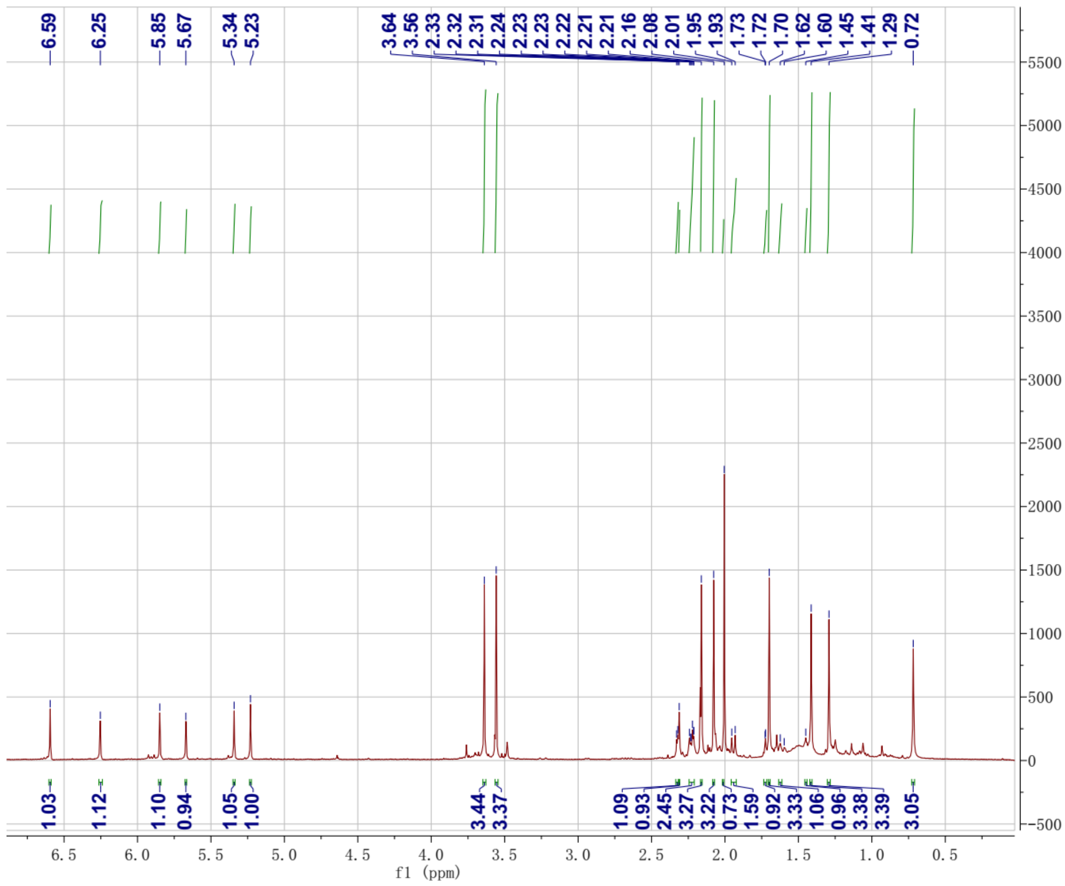

Figure S2. ^{1}H-NMR spectrum ($CDCl_3$, 500 MHz) of **1**

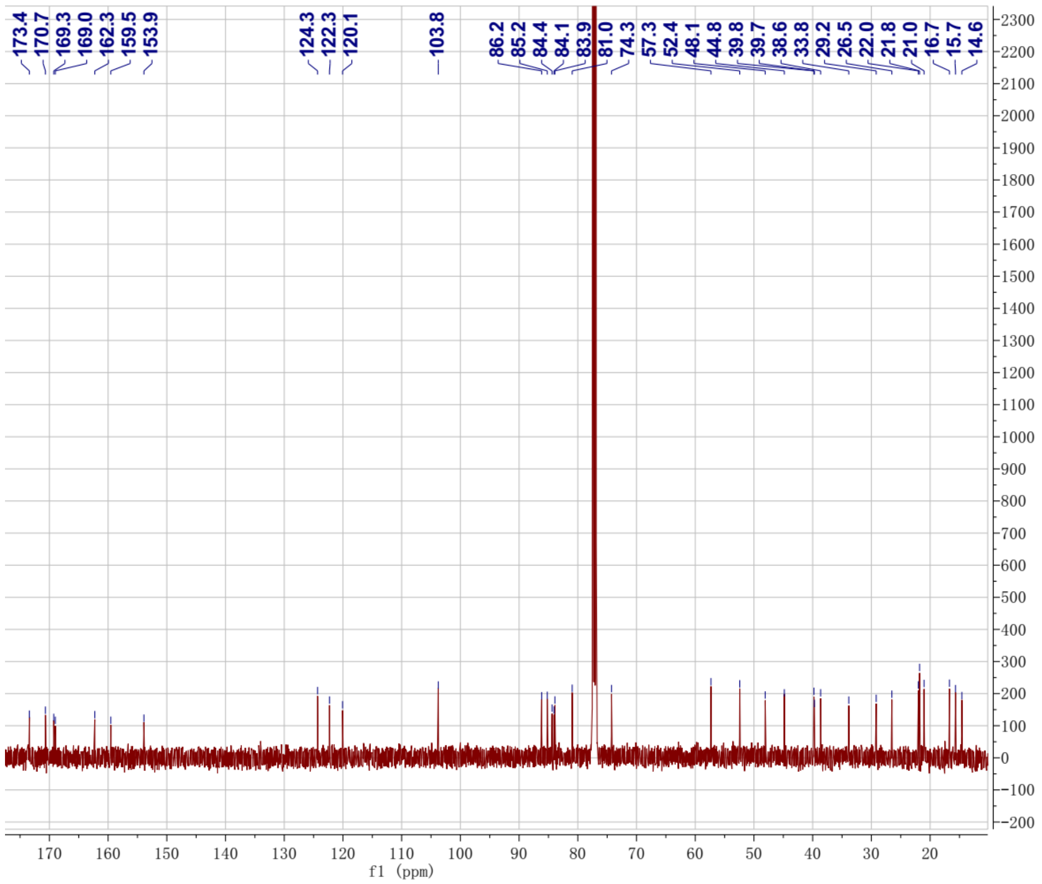

Figure S3. ^{13}C-NMR spectrum ($CDCl_3$, 125 MHz) of **1**

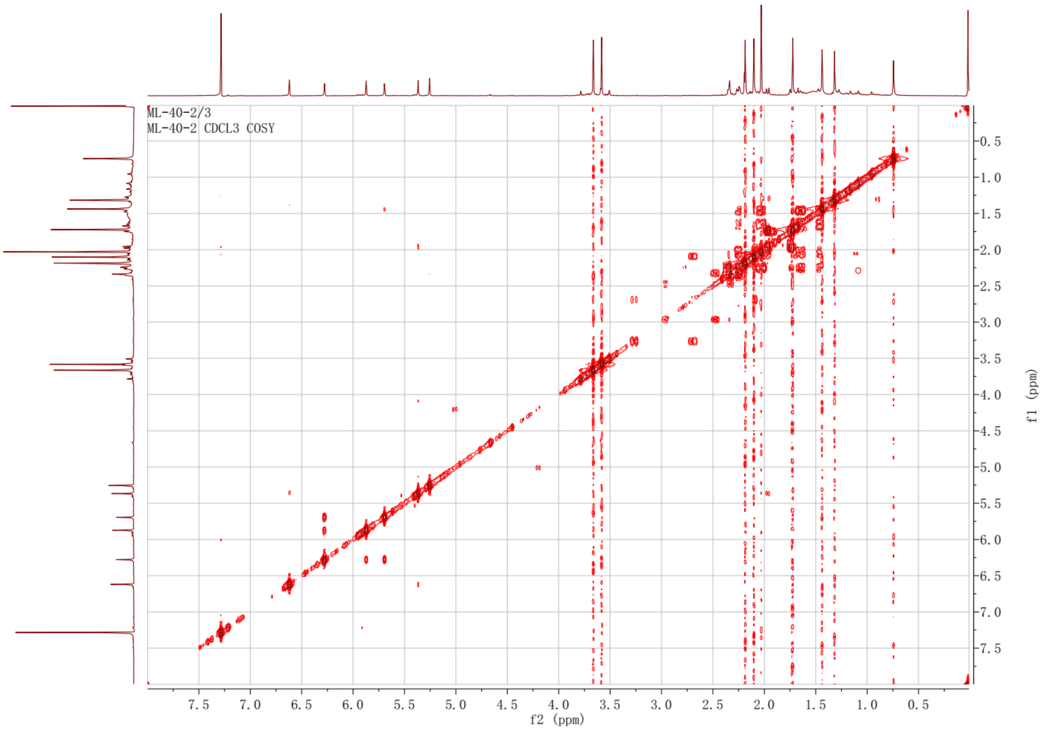

Figure S4. ^{1}H-^{1}H COSY spectrum of **1**

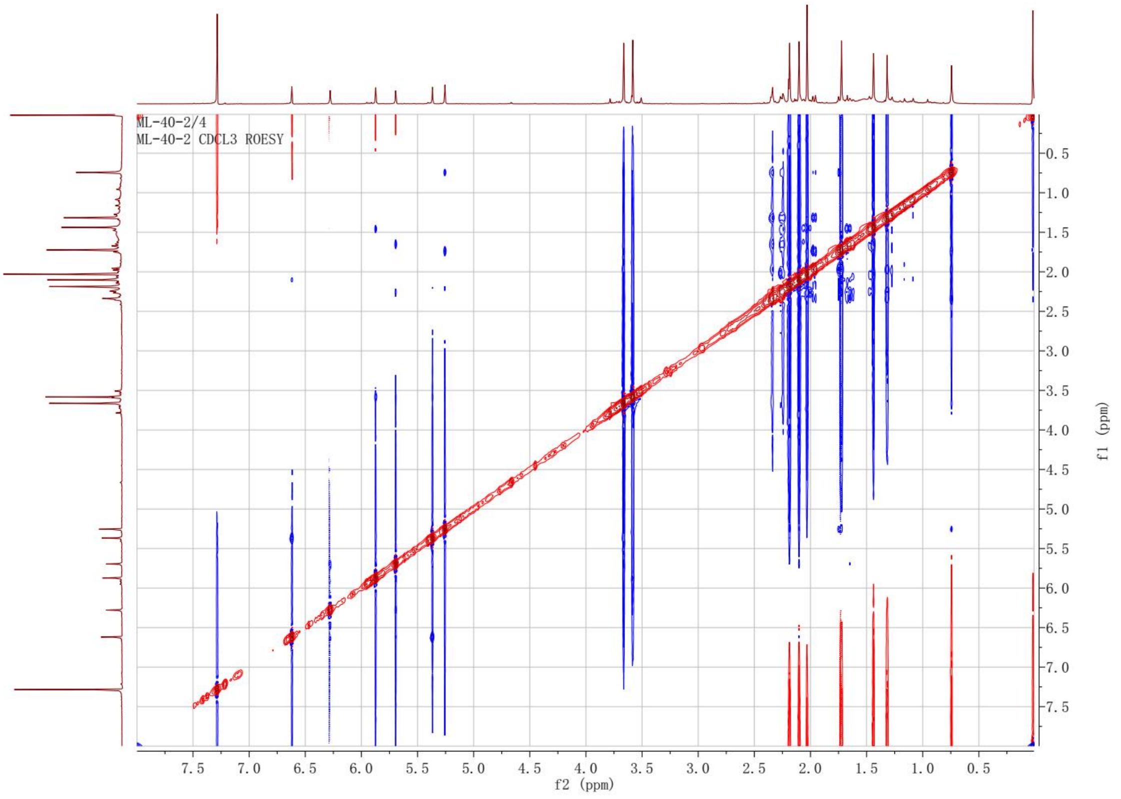

Figure S5. ROESY spectrum of **1**

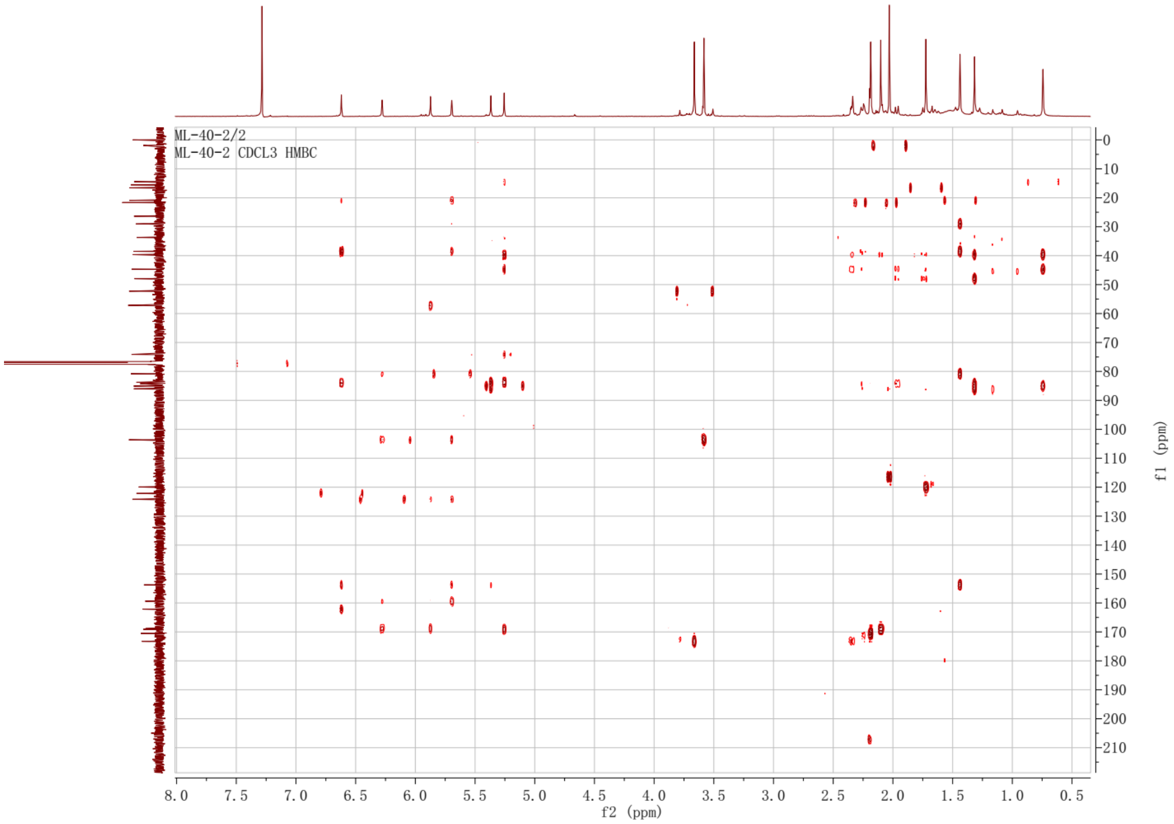

Figure S6. HMBC spectrum of **1**

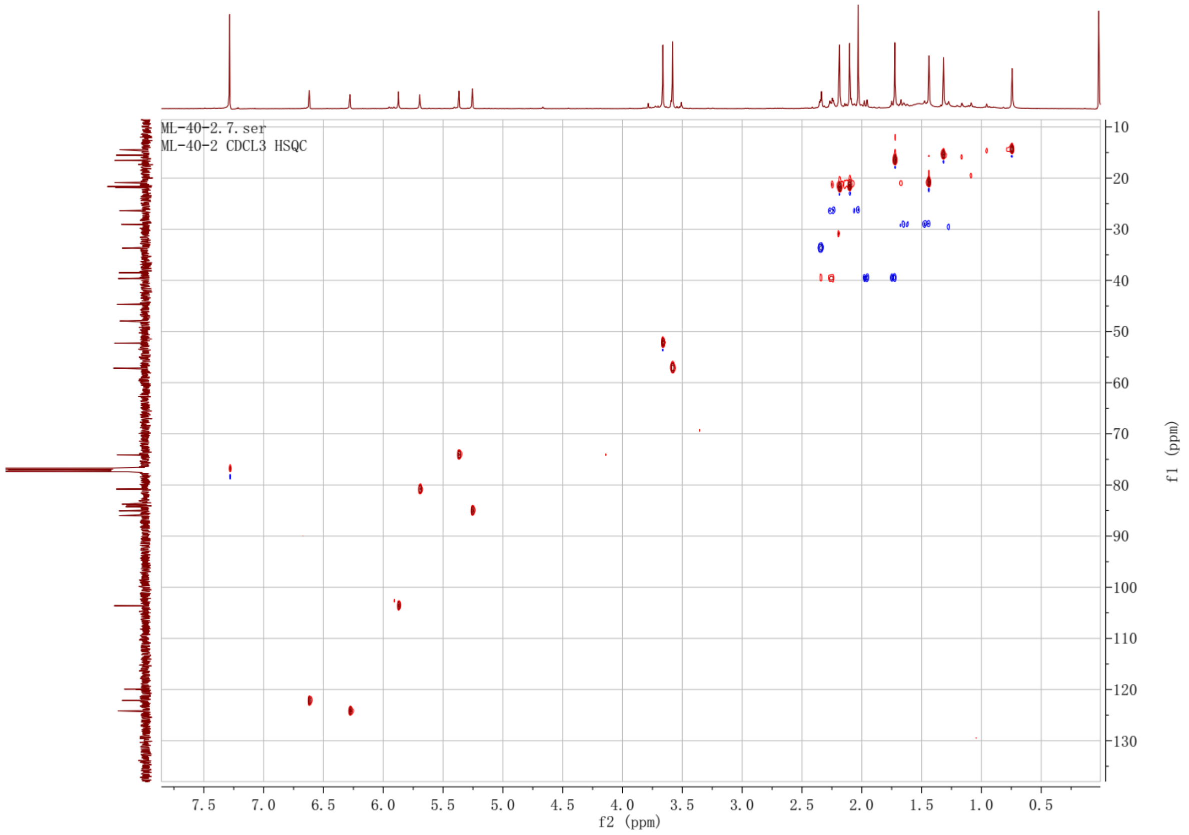

Figure S7. HSQC spectrum of **1**

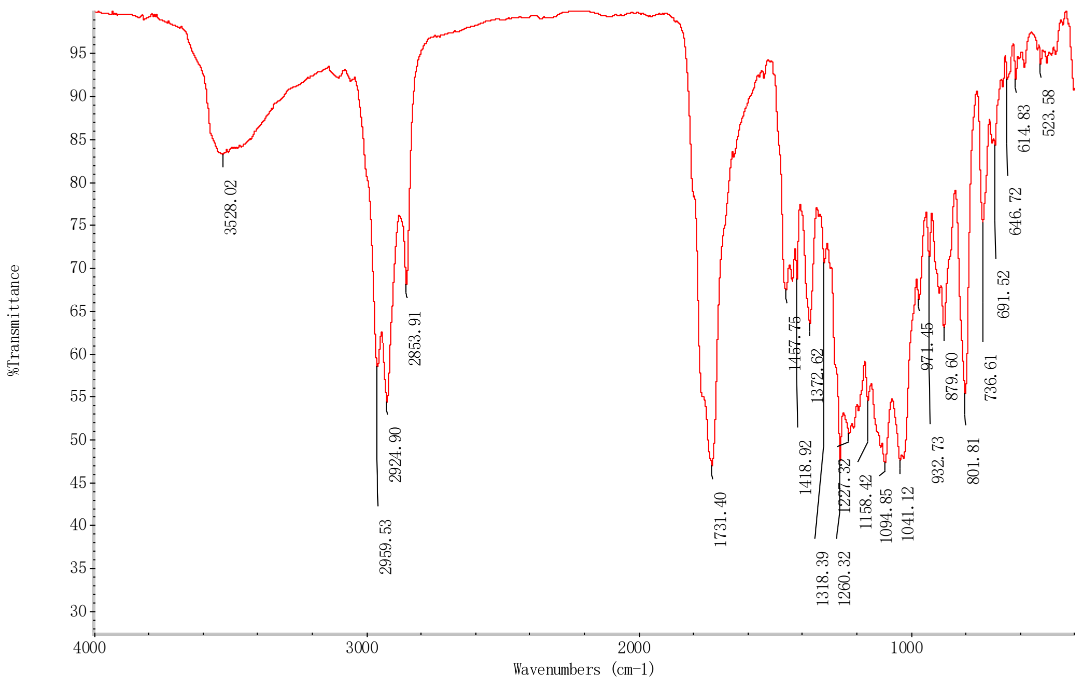

Figure S8. IR spectrum of **1**

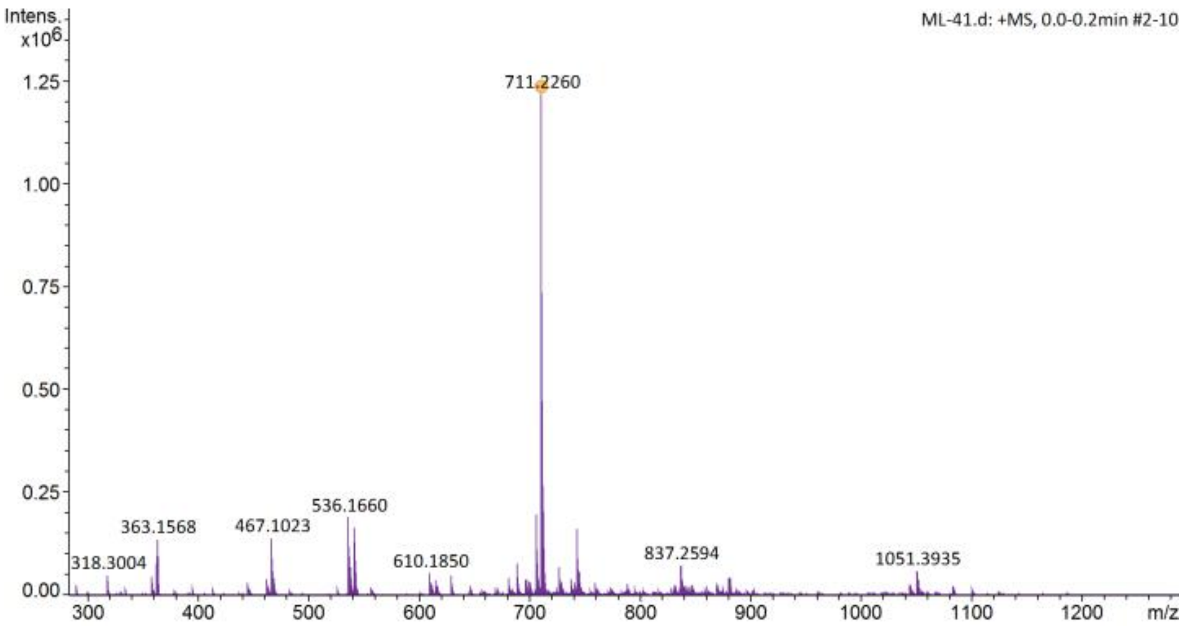

Sum Formula	Ion Formula	Meas. m/z	m/z	err [mDa]	err [ppm]
C34H40O15	C34H40NaO15	711.2260	711.2259	-0.0	-0.0

Figure S9. HR-ESI-MS spectrum of **2**

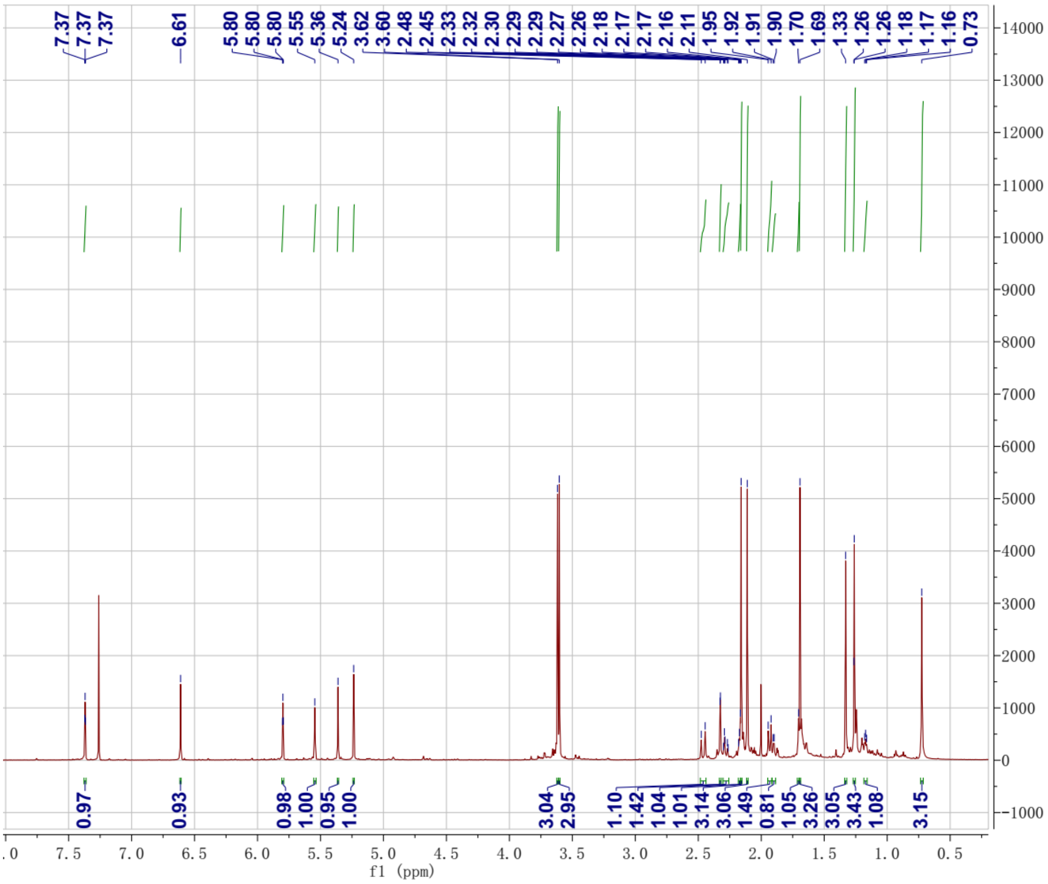

Figure S10. ^{1}H-NMR spectrum ($CDCl_3$, 500 MHz) of **2**

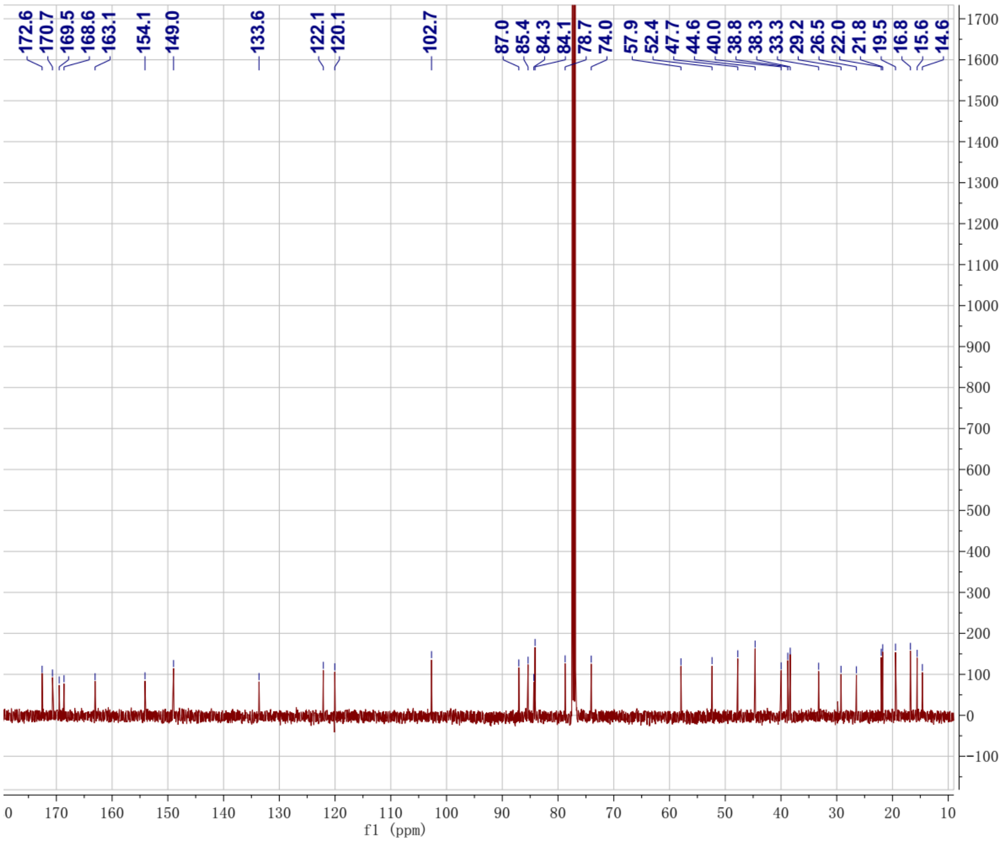

Figure S11. ^{13}C-NMR spectrum ($CDCl_3$, 125 MHz) of **2**

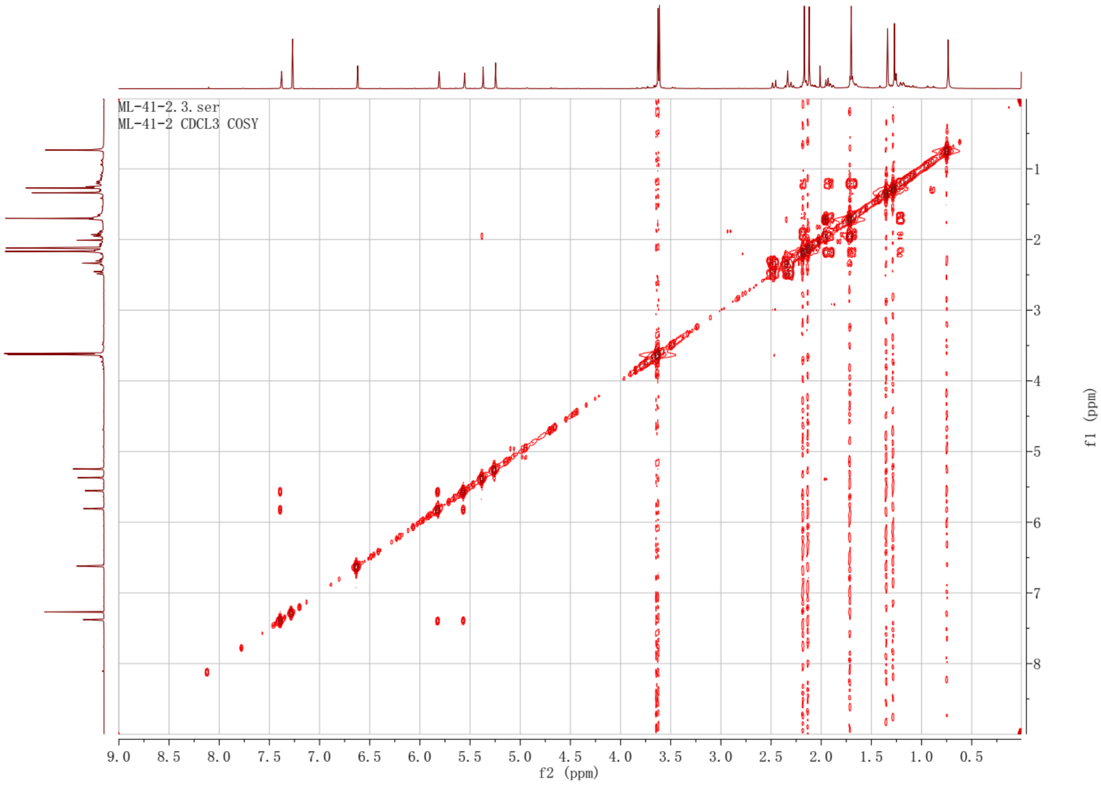

Figure S12. ^{1}H-^{1}H COSY spectrum of **2**

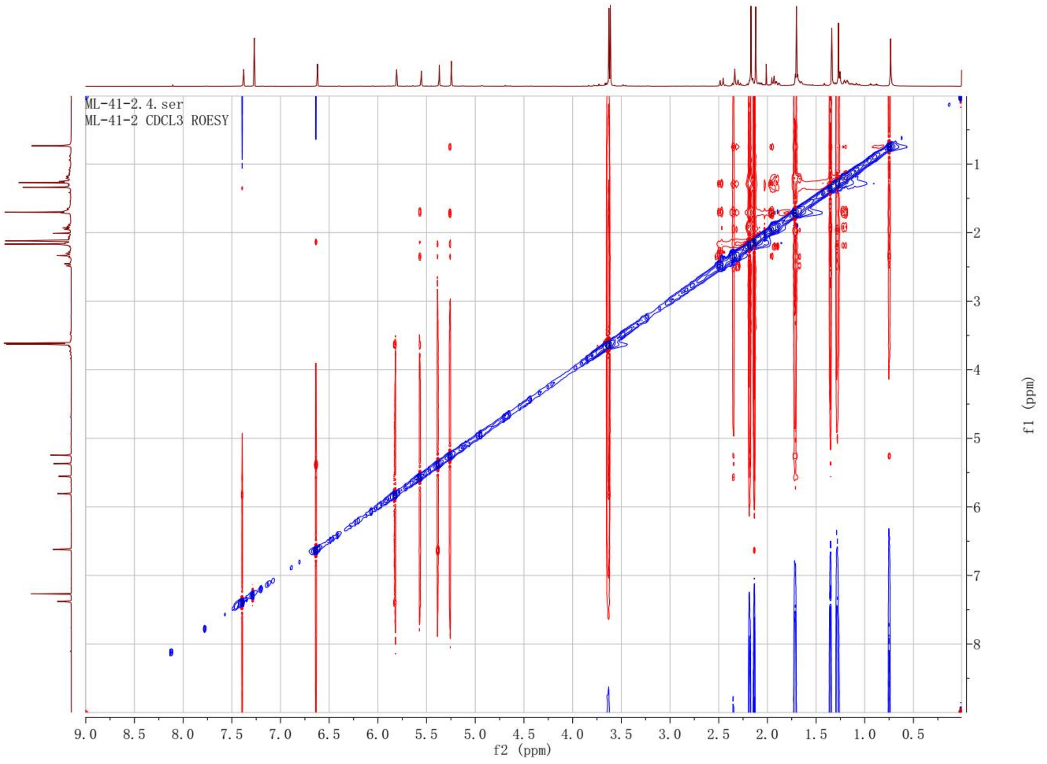

Figure S13. ROESY spectrum of **2**

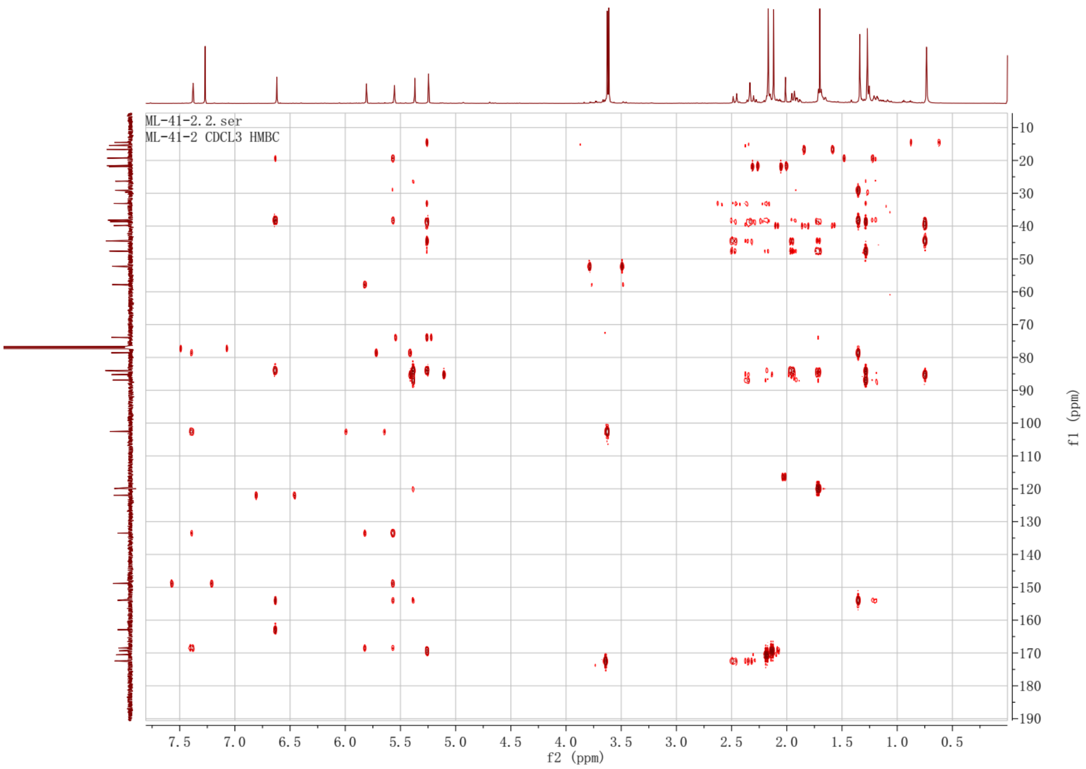

Figure S14. HMBC spectrum of **2**

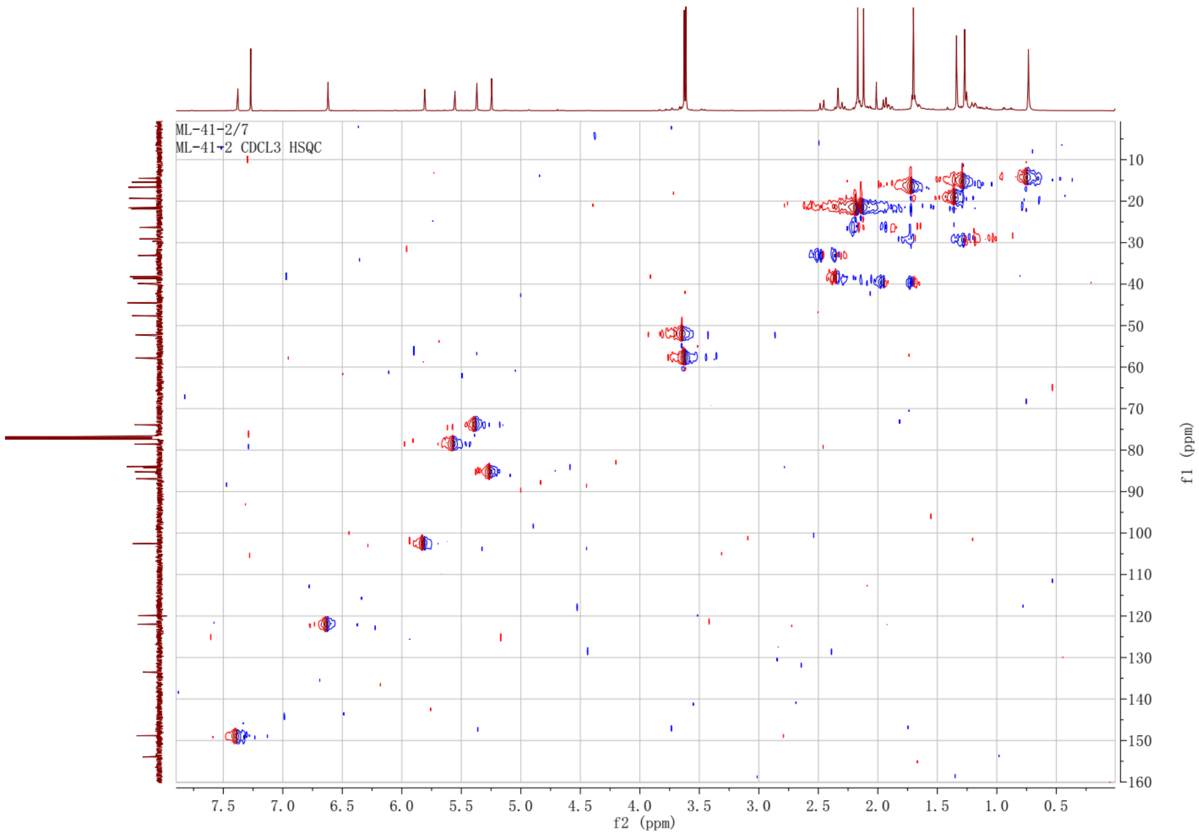

Figure S15. HSQC spectrum of **2**

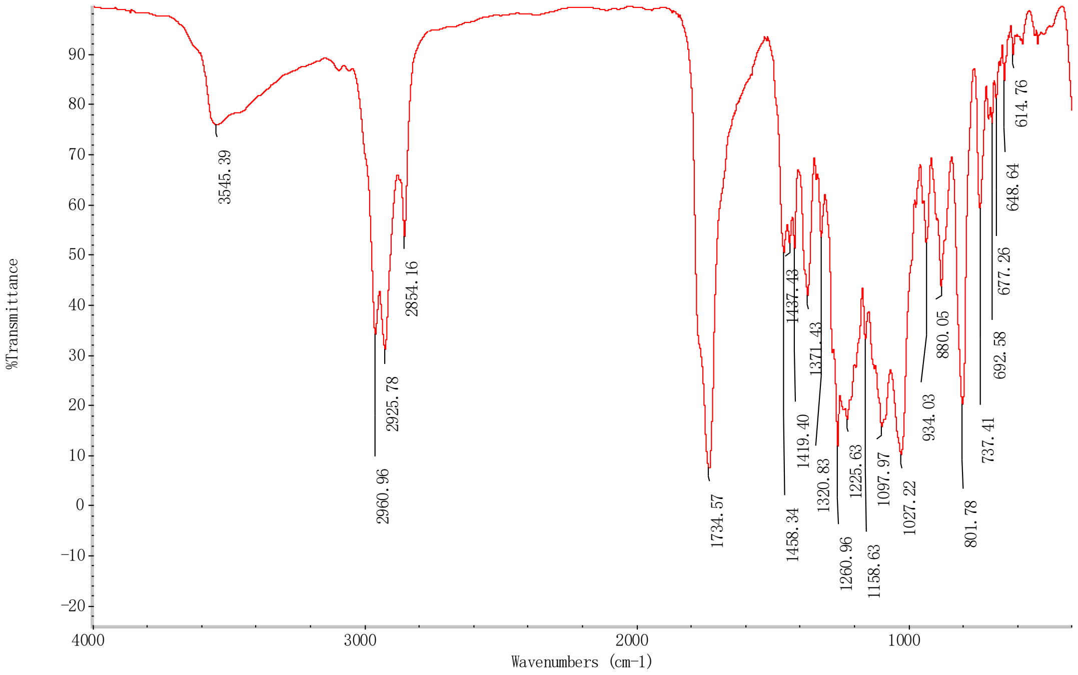

Figure S16. IR spectrum of **2**

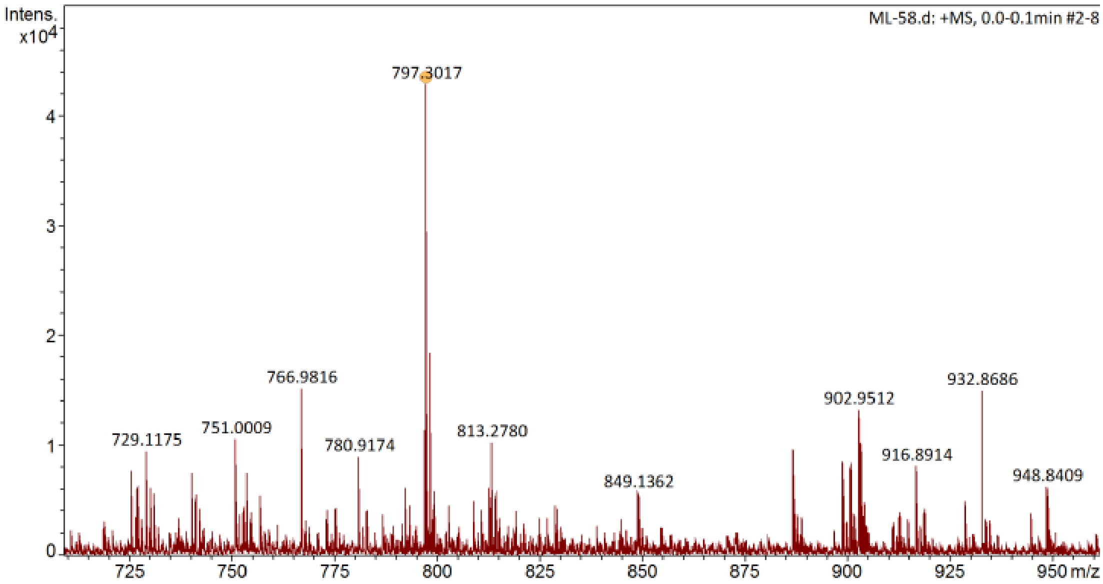

Sum Formula	Ion Formula	Meas. m/z	m/z	err [mDa]	err [ppm]
C39H50O16	C39H50NaO16	797.3017	797.2991	-2.6	-3.3

Figure S17. HR-ESI-MS spectrum of **3**

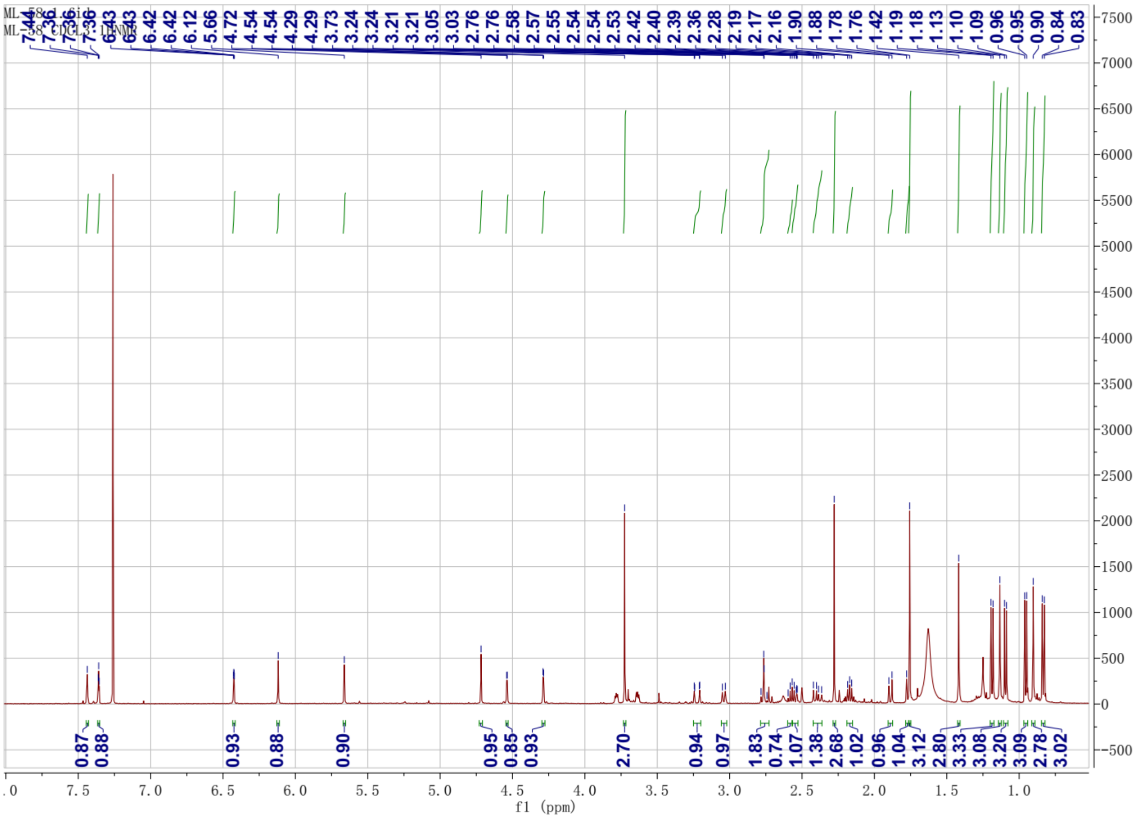

Figure S18. ^{1}H-NMR spectrum ($CDCl_3$, 500 MHz) of **3**

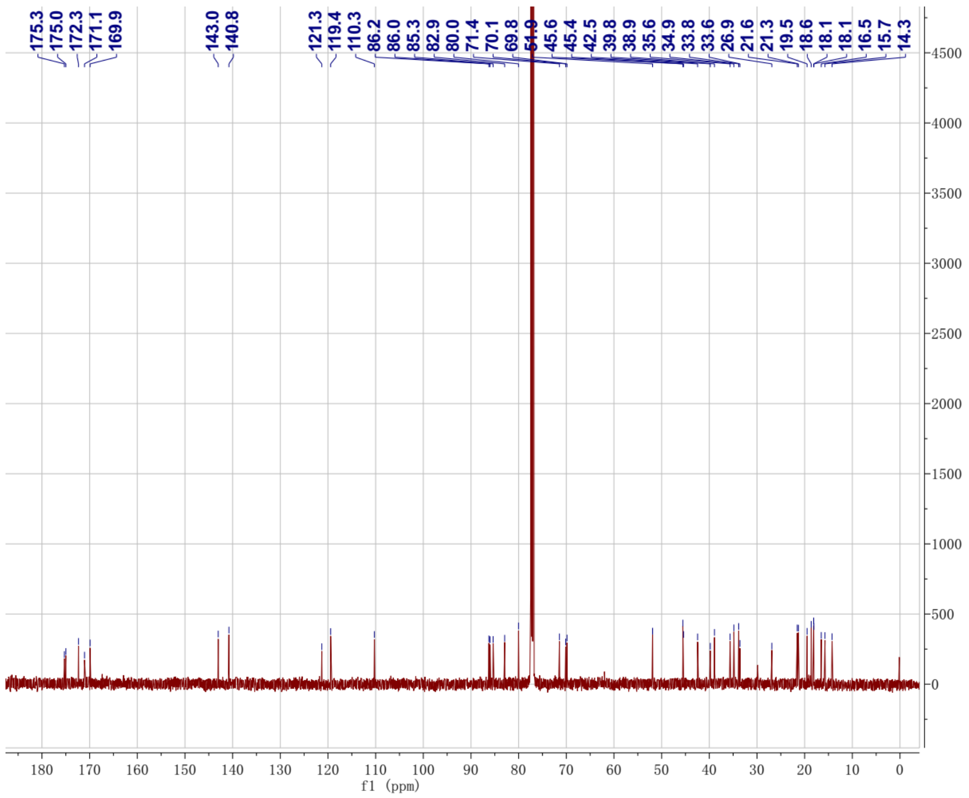

Figure S19. ^{13}C-NMR spectrum ($CDCl_3$, 125 MHz) of **3**

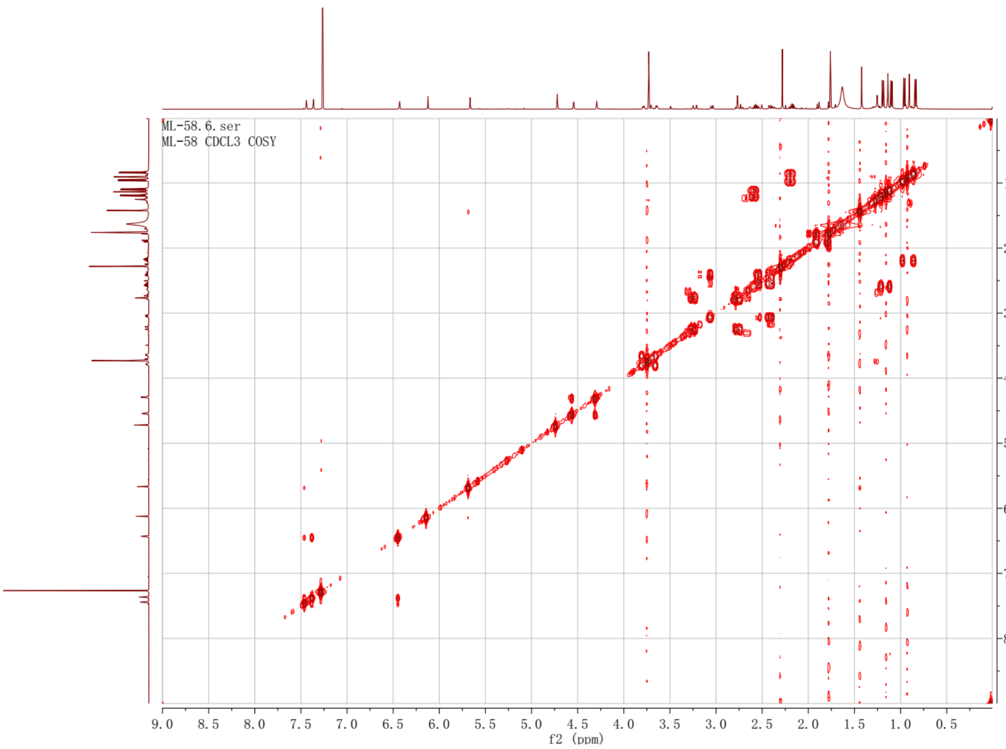

Figure S20. ^{1}H-^{1}H COSY spectrum of **3**

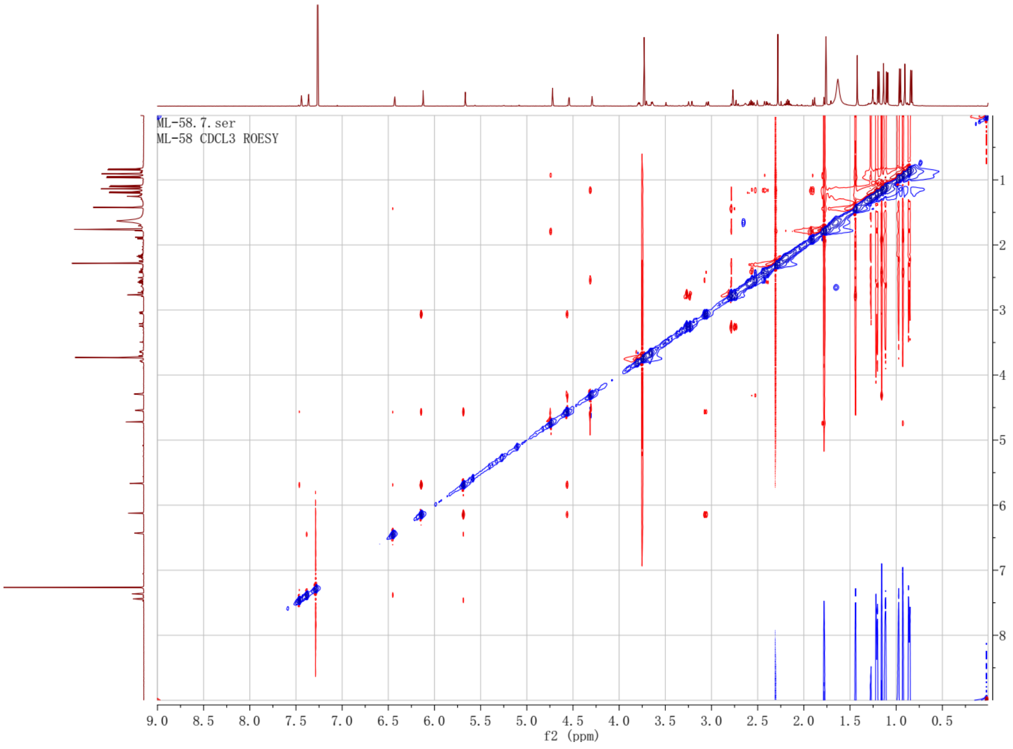

Figure S21. ROESY spectrum of **3**

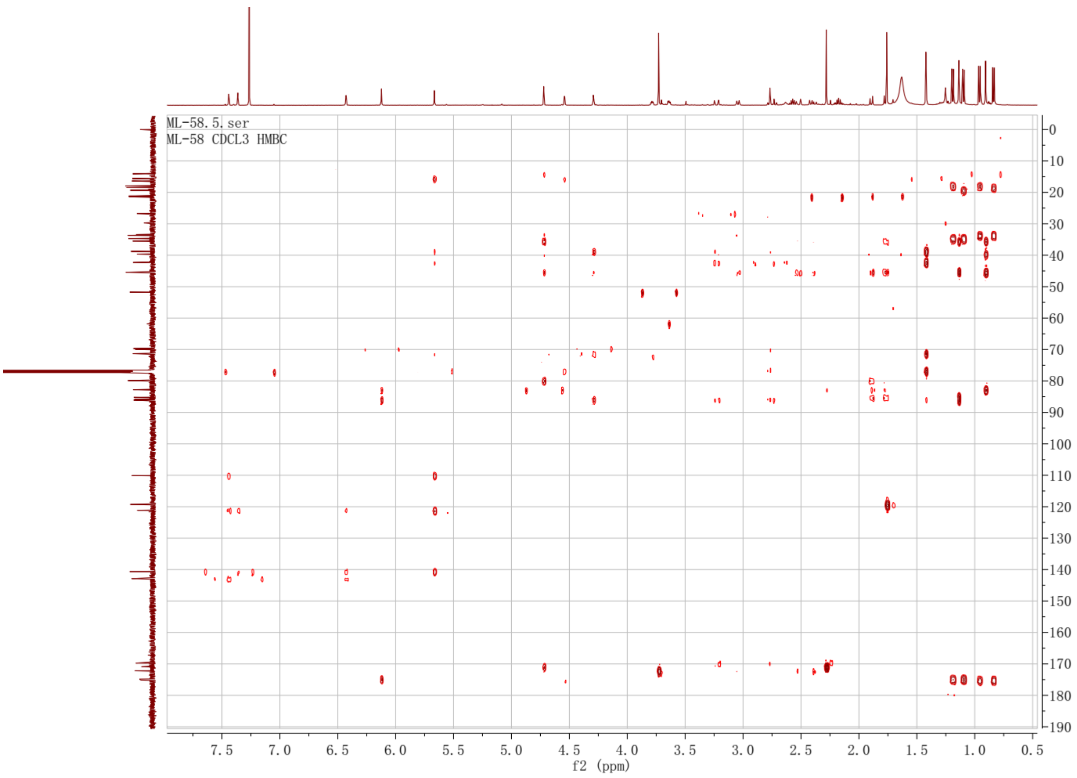

Figure S22. HMBC spectrum of **3**

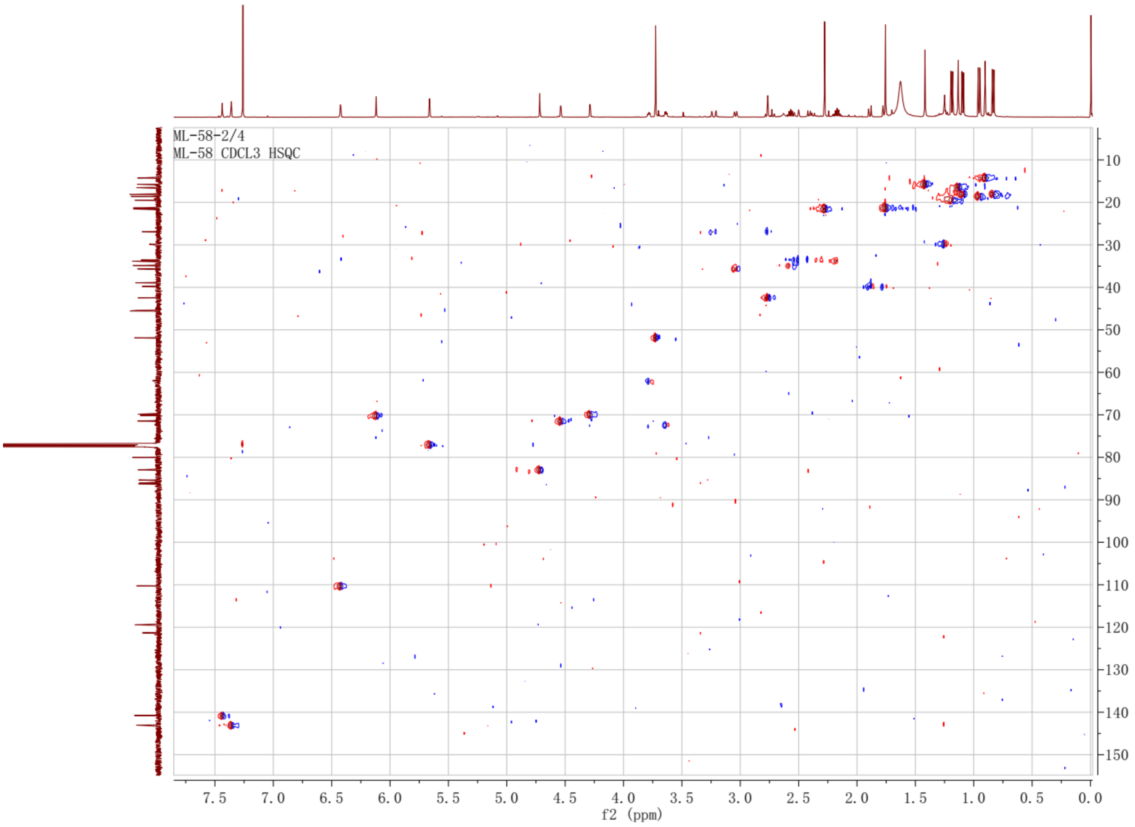

Figure S23. HSQC spectrum of **3**

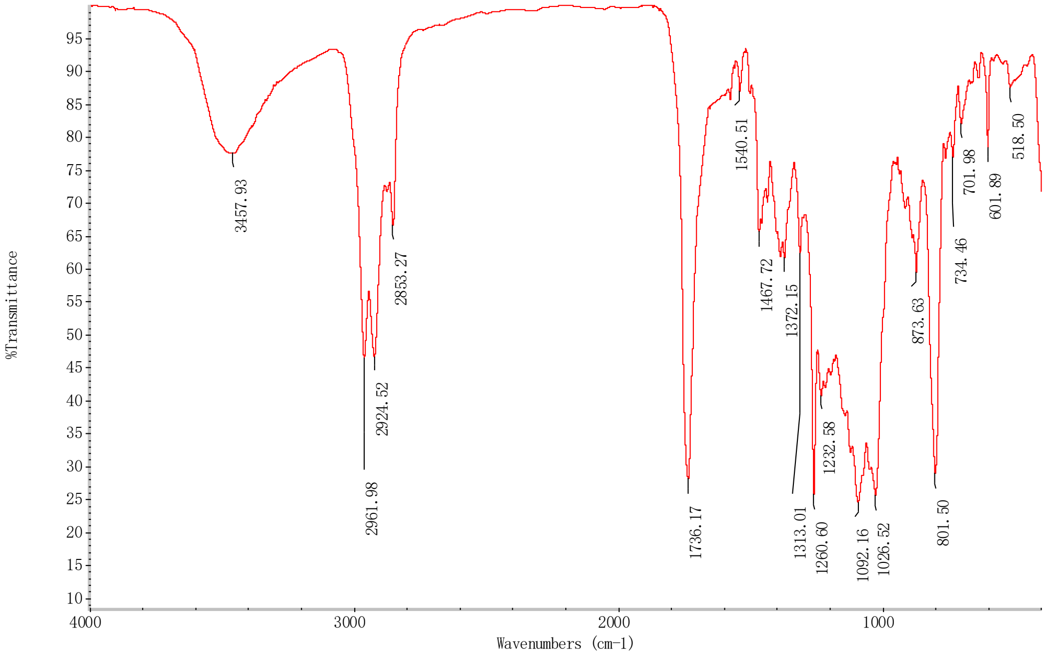

Figure S24. IR spectrum of **3**

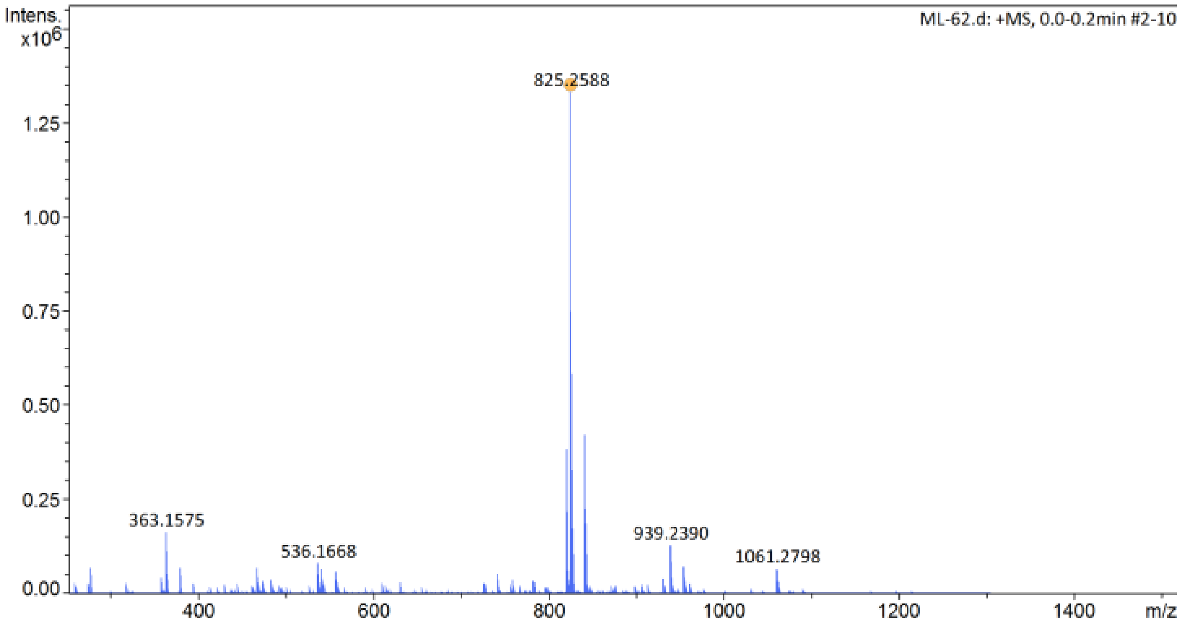

Sum Formula	Ion Formula	Meas. m/z	m/z	err [mDa]	err [ppm]
C39H46O18	C39H46NaO18	825.2588	825.2576	-1.2	-1.4

Figure S25. HR-ESI-MS spectrum of **4**

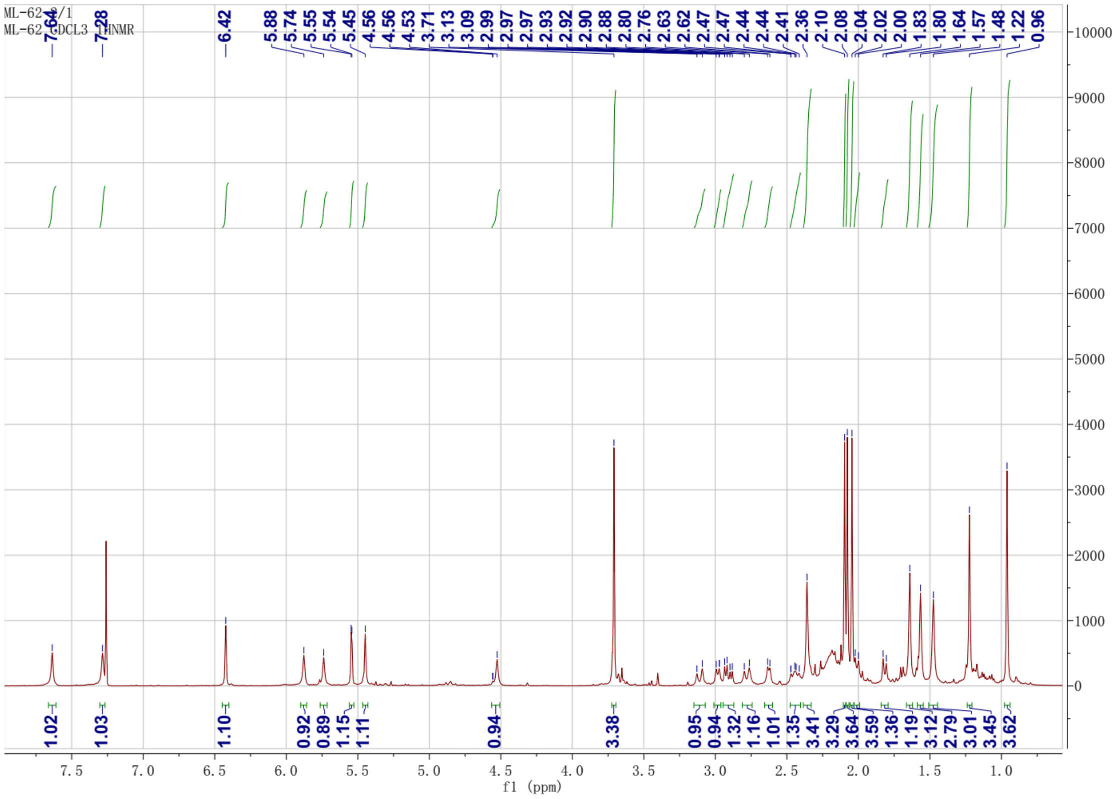

Figure S26. ^{1}H-NMR spectrum ($CDCl_3$, 500 MHz) of **4**

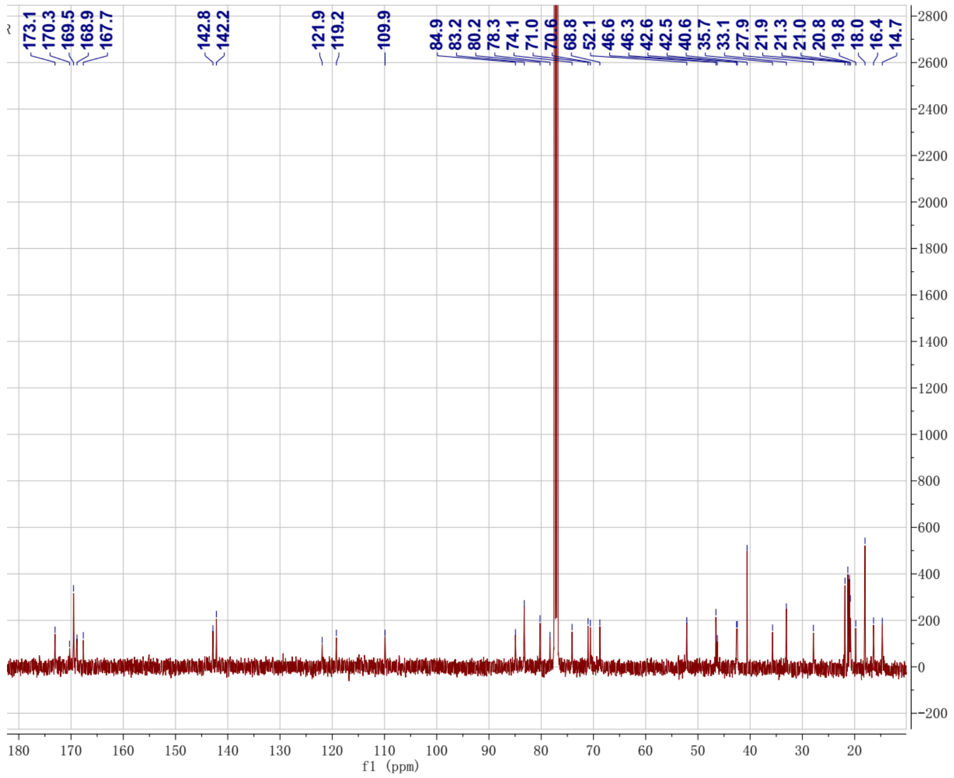

Figure S27. ^{13}C-NMR spectrum ($CDCl_3$, 125 MHz) of **4**

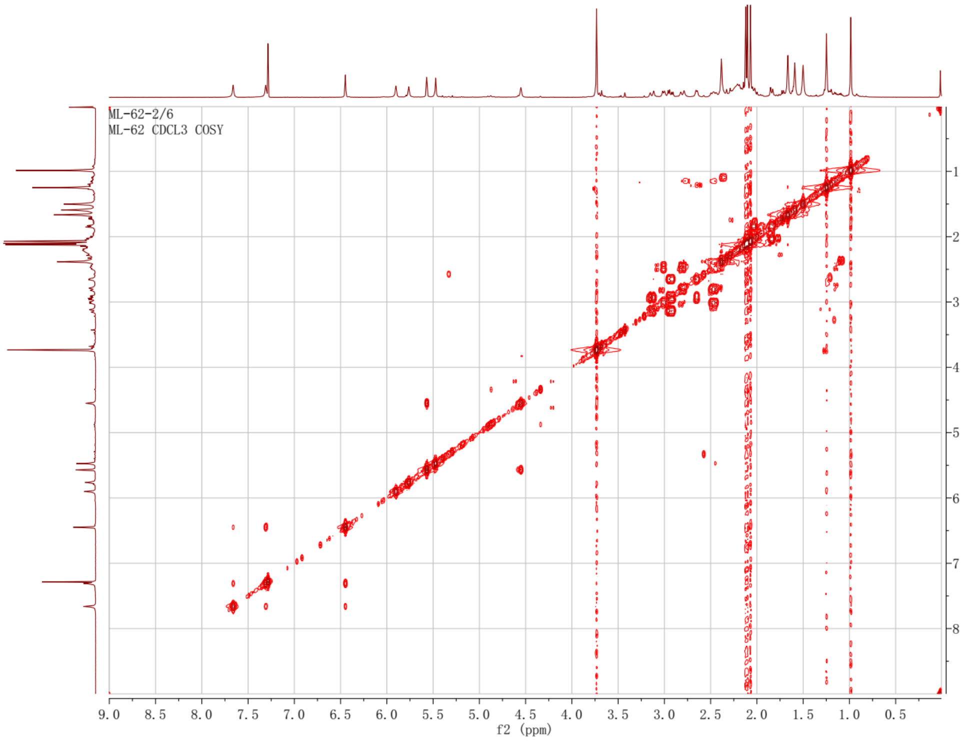

Figure S28. ^{1}H-^{1}H COSY spectrum of **4**

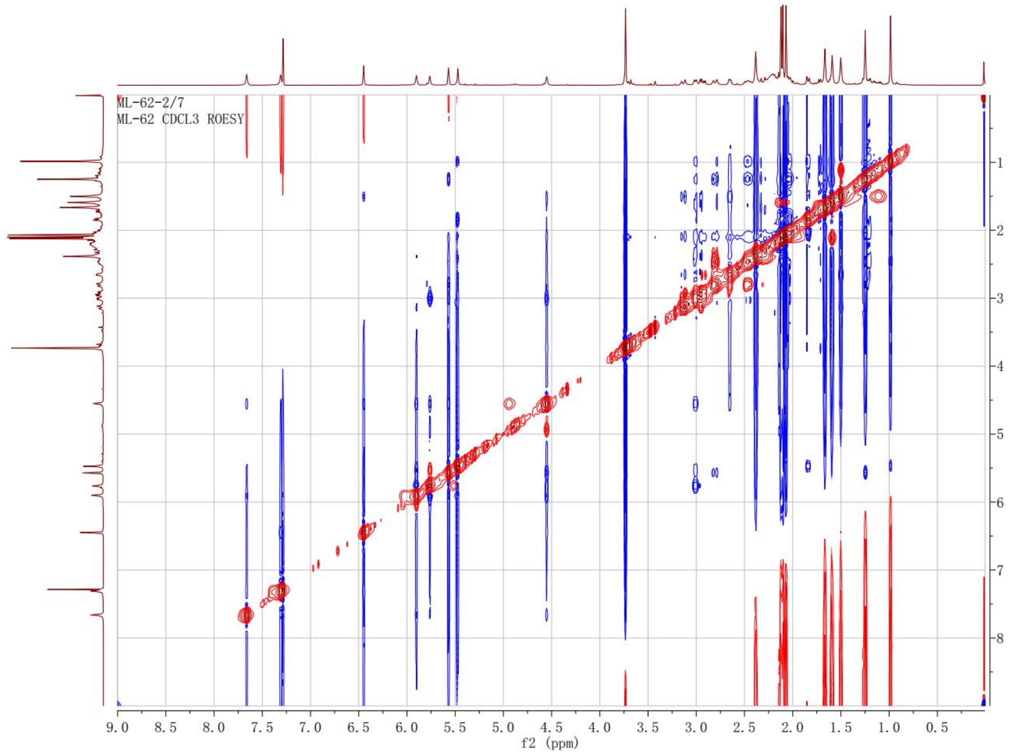

Figure S29. ROESY spectrum of **4**

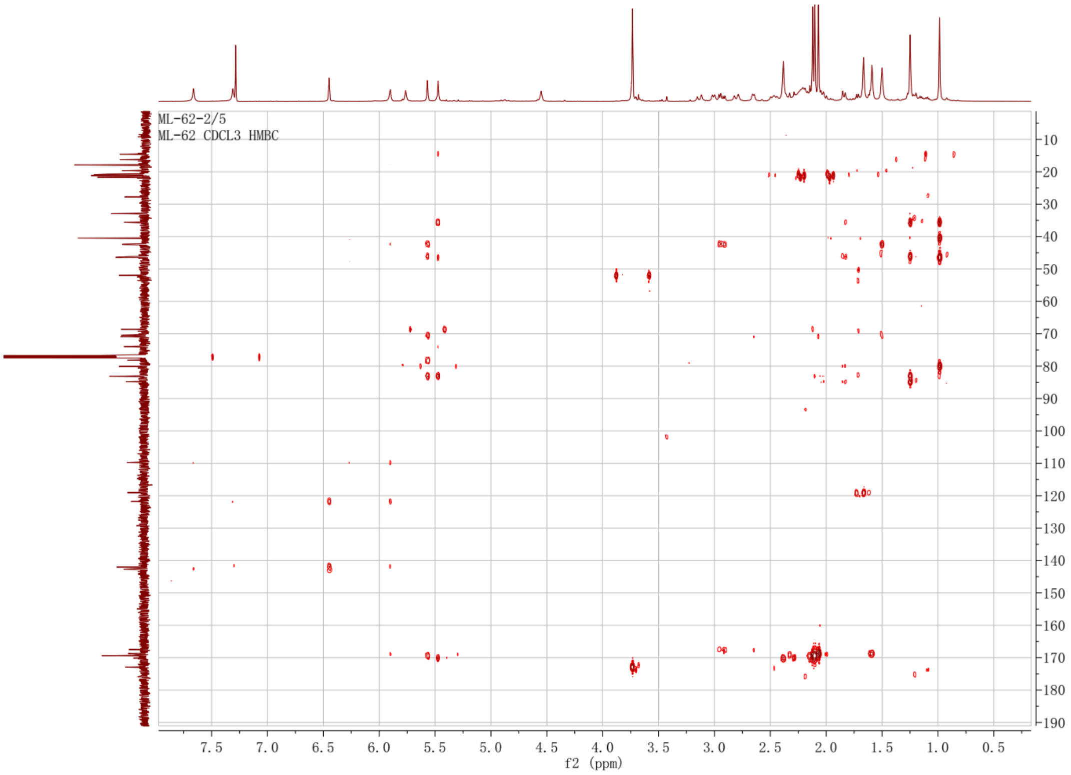

Figure S30. HMBC spectrum of **4**

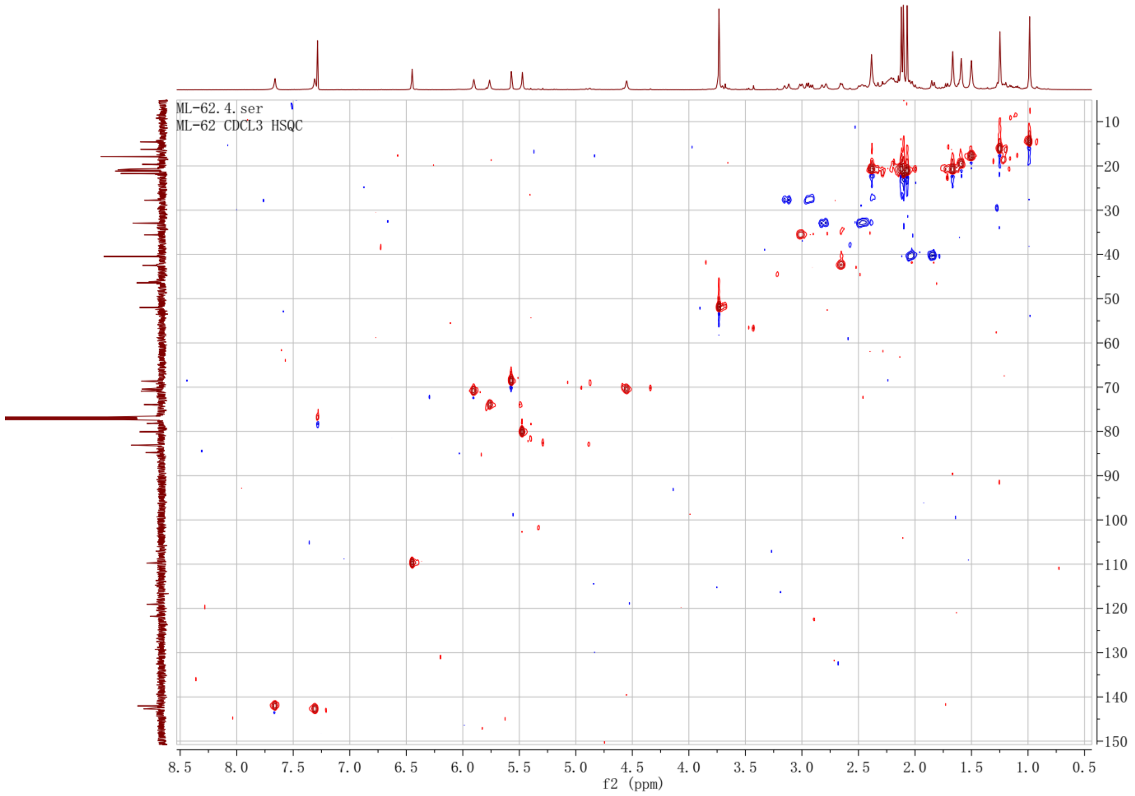

Figure S31. HSQC spectrum of **4**

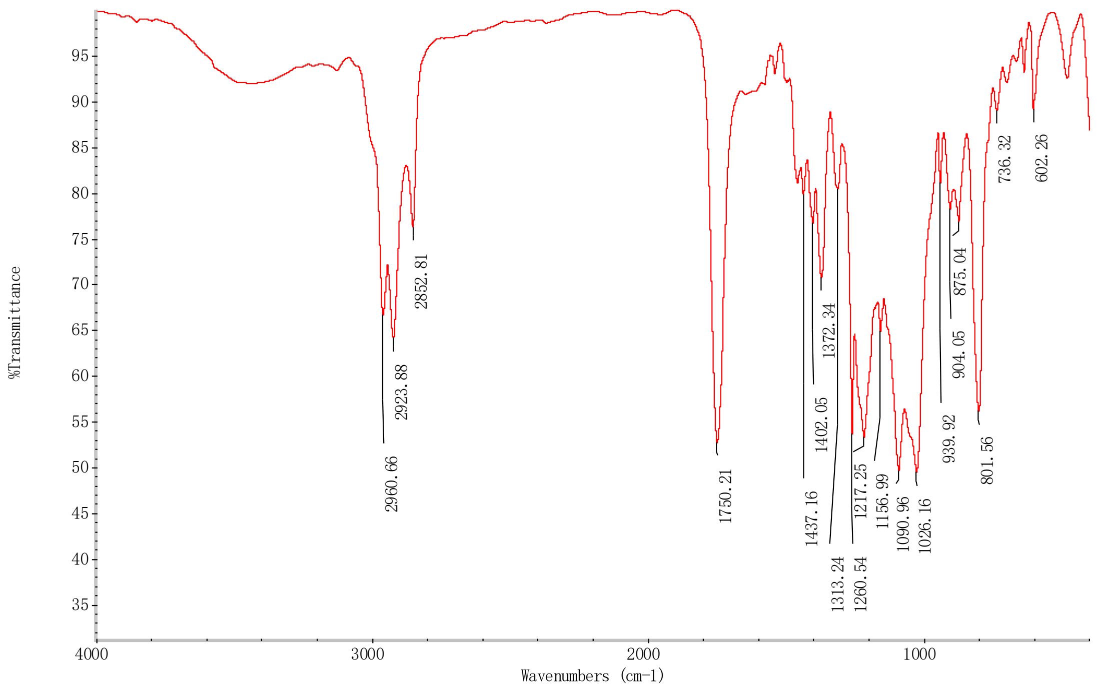

Figure S32. IR spectrum of **4**

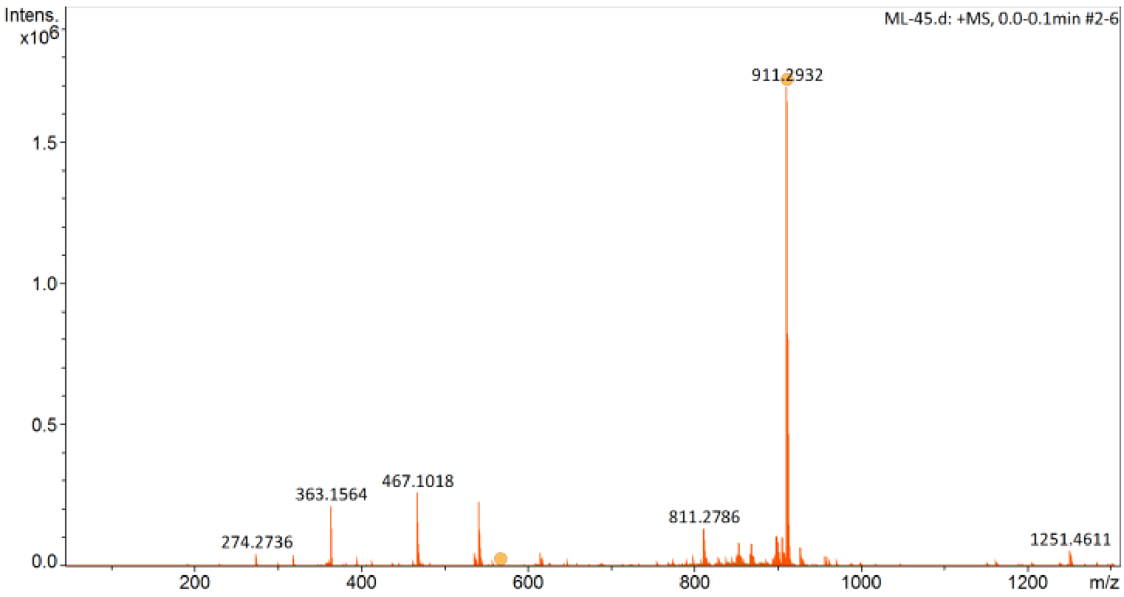

Sum Formula	Ion Formula	Meas. m/z	m/z	err [mDa]	err [ppm]
C43H52O20	C43H52NaO20	911.2932	911.2944	1.2	1.4

Figure S33. HR-ESI-MS spectrum of **5**

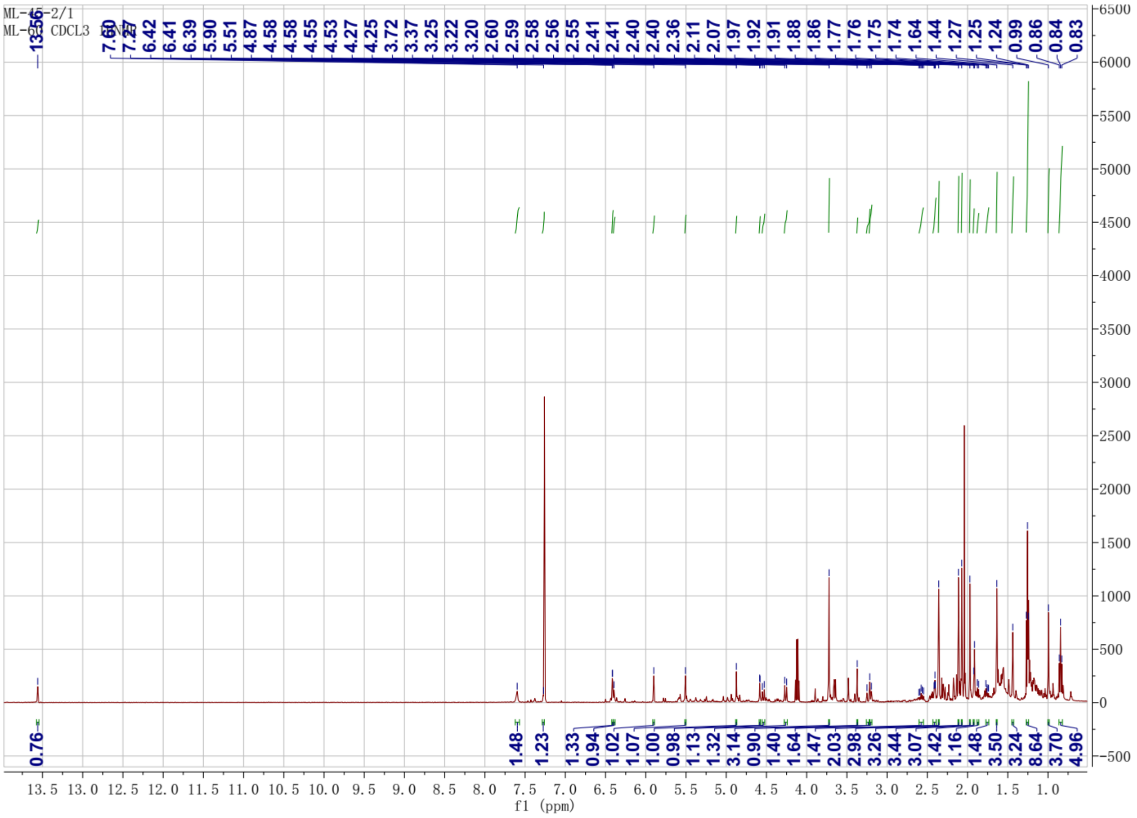

Figure S34. ^{1}H-NMR spectrum ($CDCl_3$, 500 MHz) of **5**

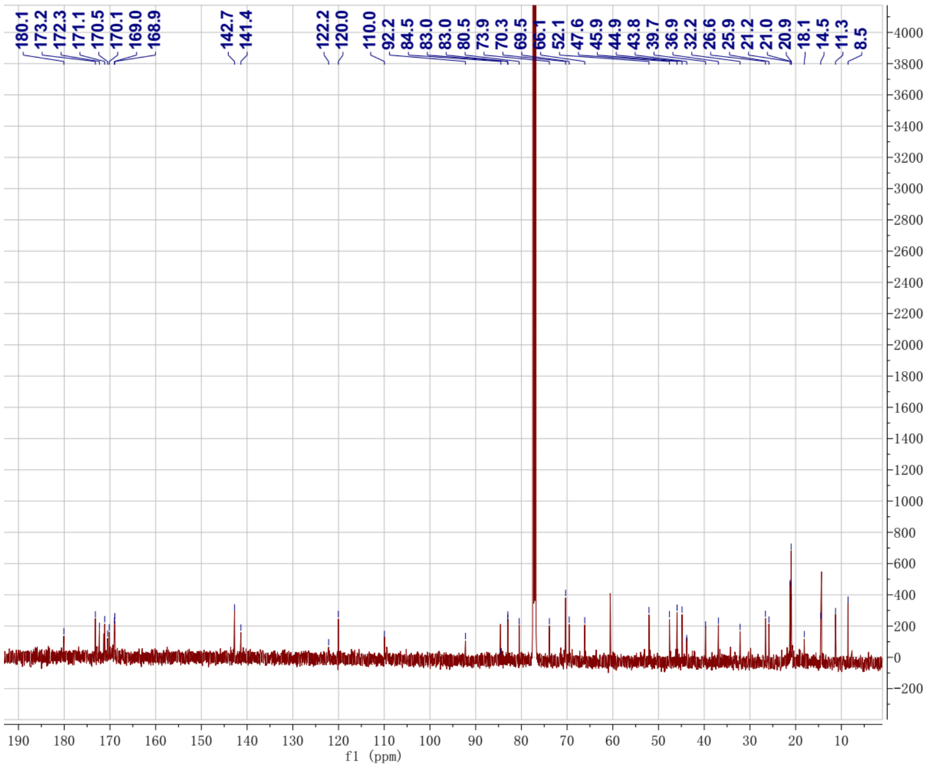

Figure S35. ^{13}C-NMR spectrum ($CDCl_3$, 125 MHz) of **5**

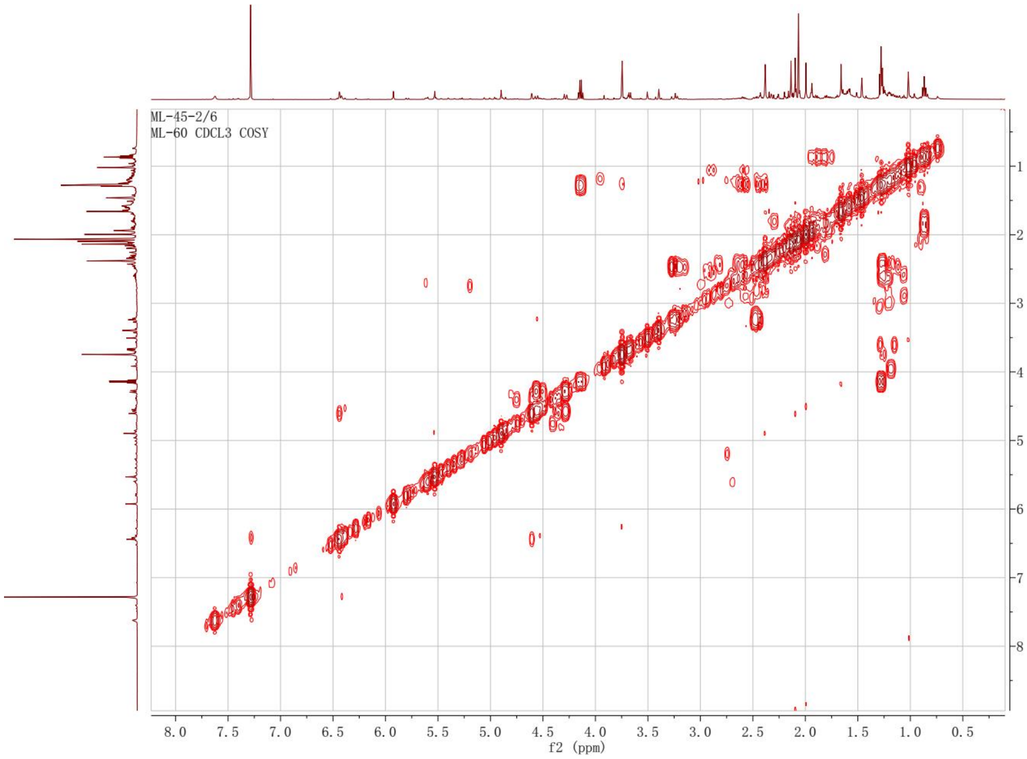

Figure S36. ^{1}H-^{1}H COSY spectrum of **5**

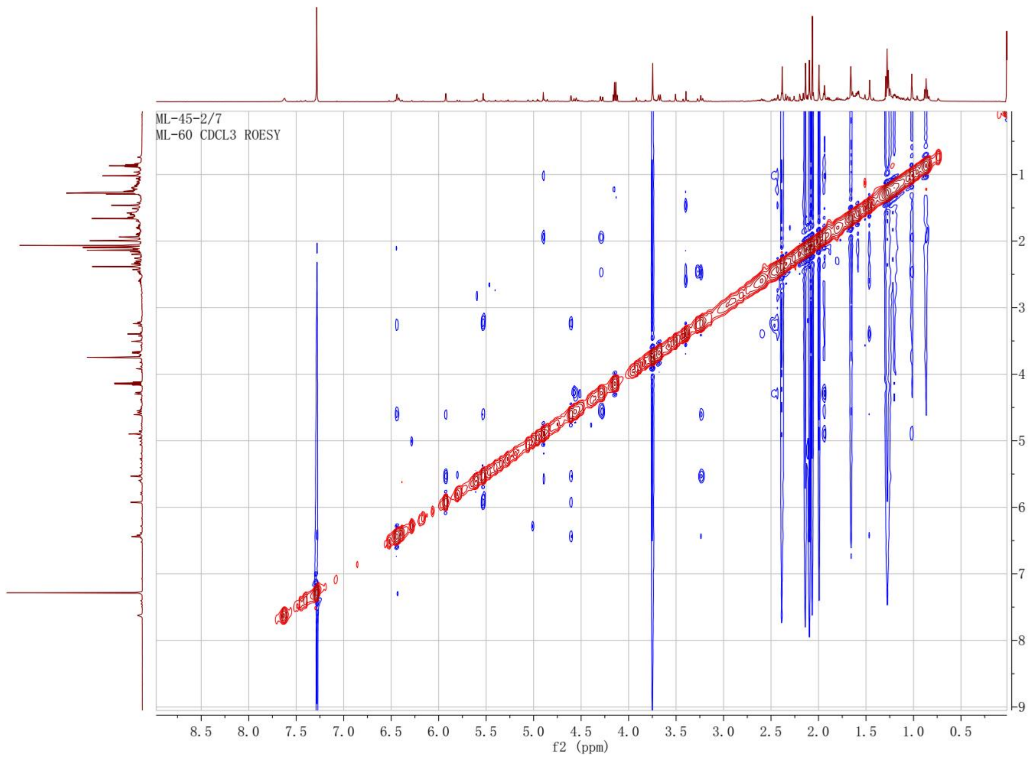

Figure S37. ROESY spectrum of **5**

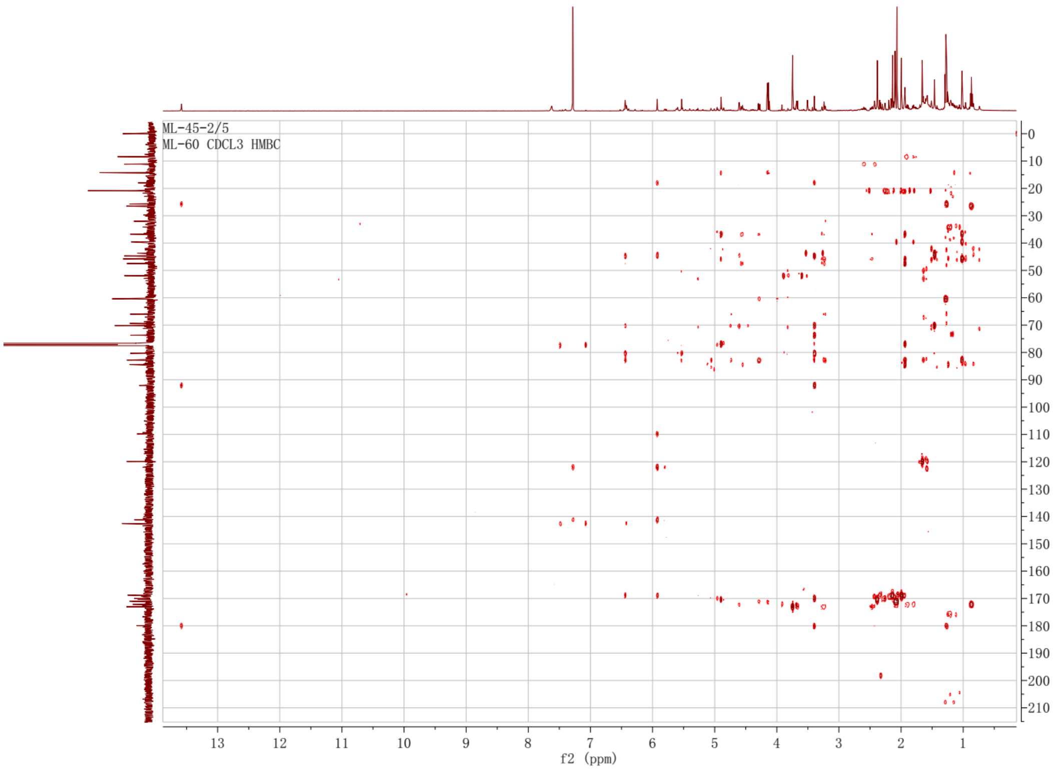

Figure S38. HMBC spectrum of **5**

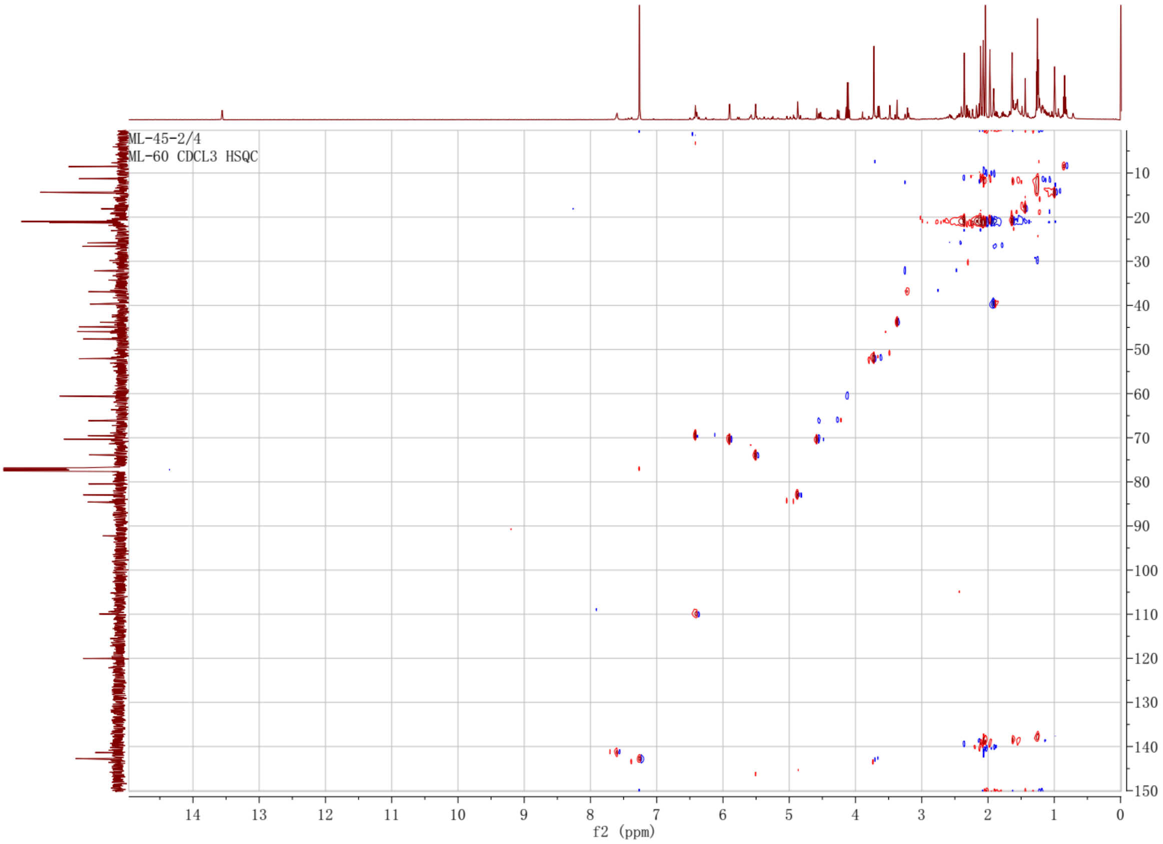

Figure S39. HSQC spectrum of **5**

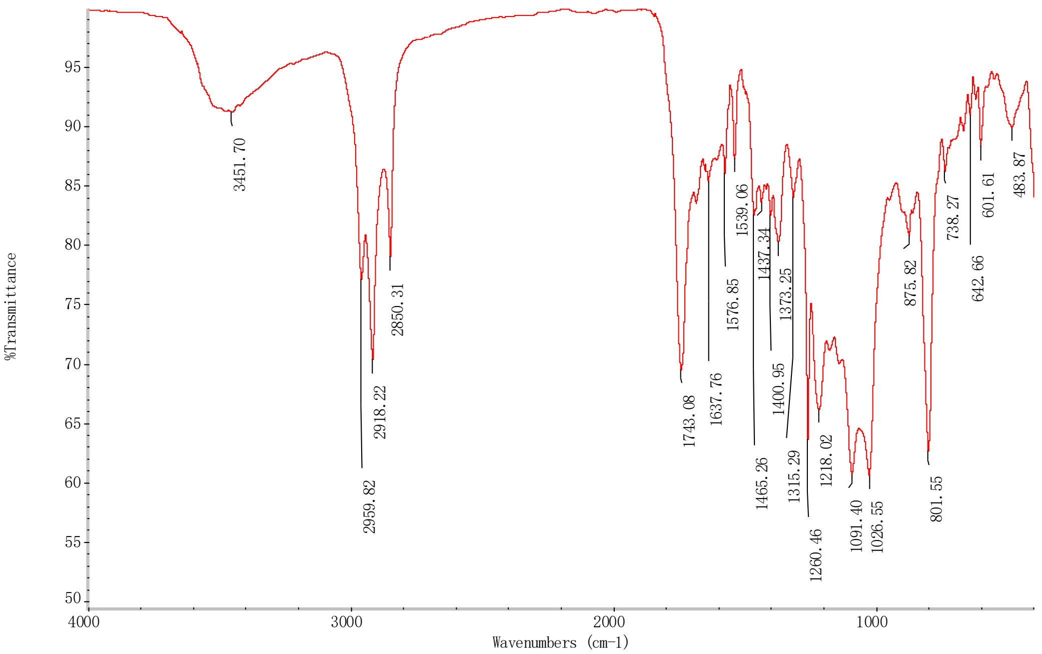

Figure S40. IR spectrum of **5**

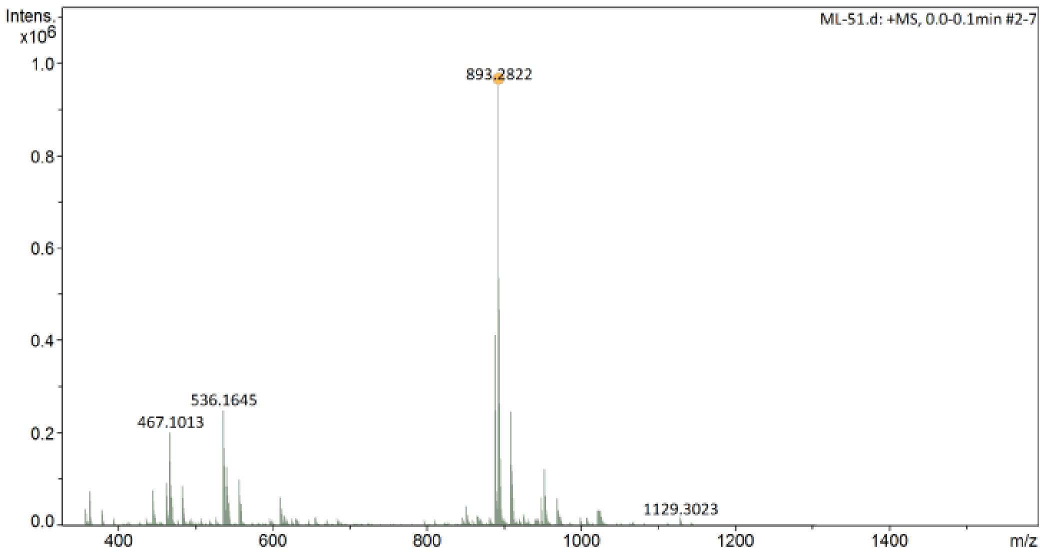

Sum Formula	Ion Formula	Meas. m/z	m/z	err [mDa]	err [ppm]
C43H50O19	C43H50NaO19	893.2822	893.2839	1.7	1.9

Figure S41. HR-ESI-MS spectrum of **6**

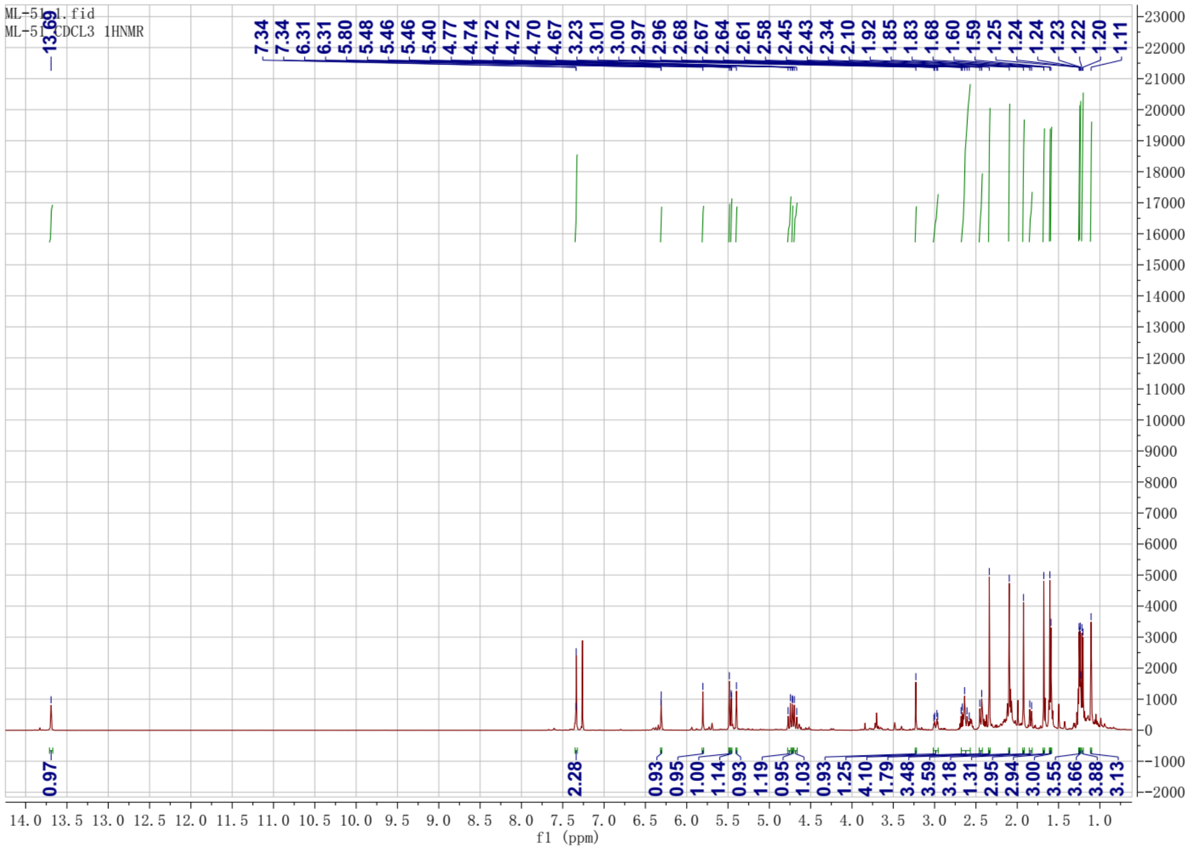

Figure S42. ^{1}H-NMR spectrum ($CDCl_3$, 500 MHz) of **6**

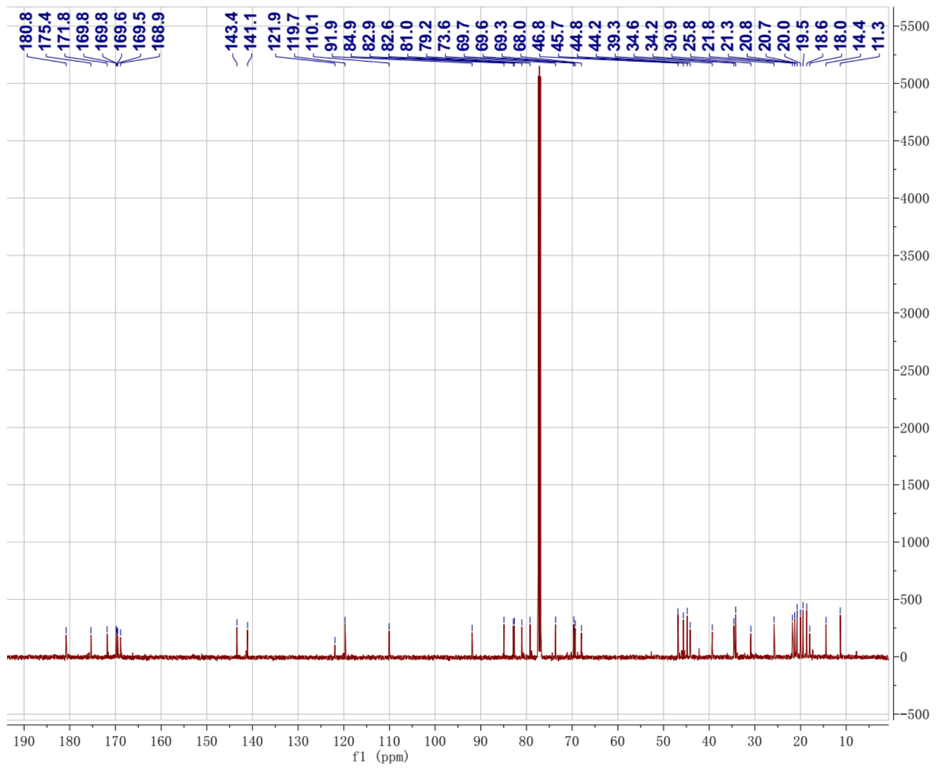

Figure S43. ^{13}C-NMR spectrum ($CDCl_3$, 125 MHz) of **6**

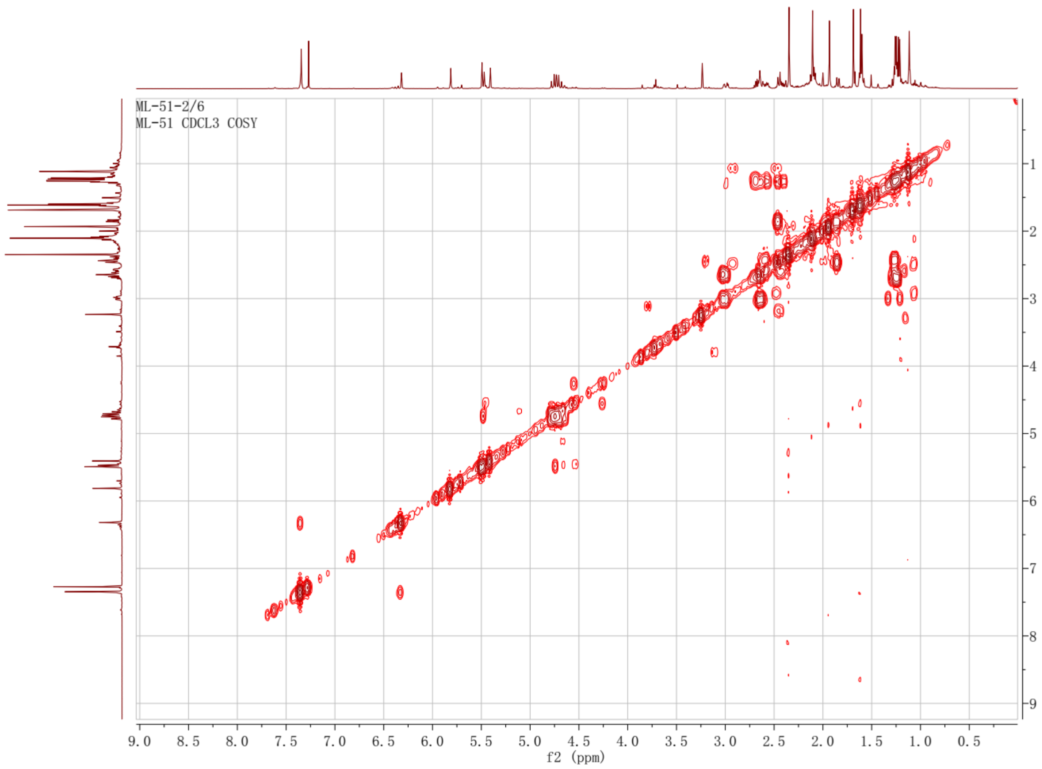

Figure S44. ^{1}H-^{1}H COSY spectrum of **6**

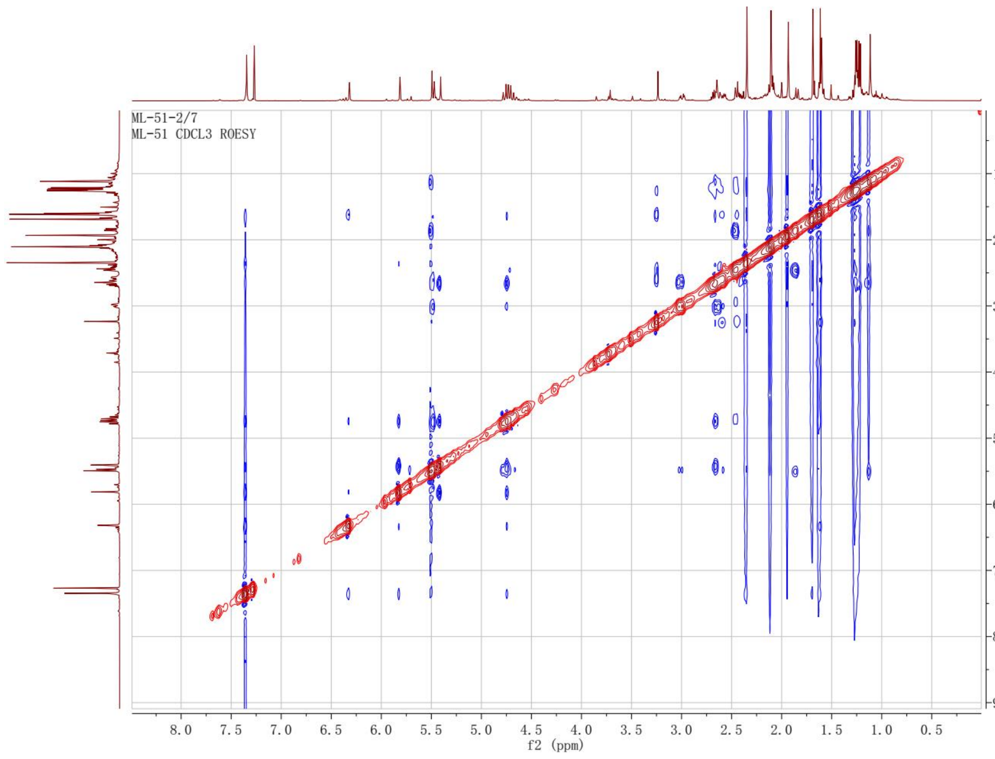

Figure S45. ROESY spectrum of **6**

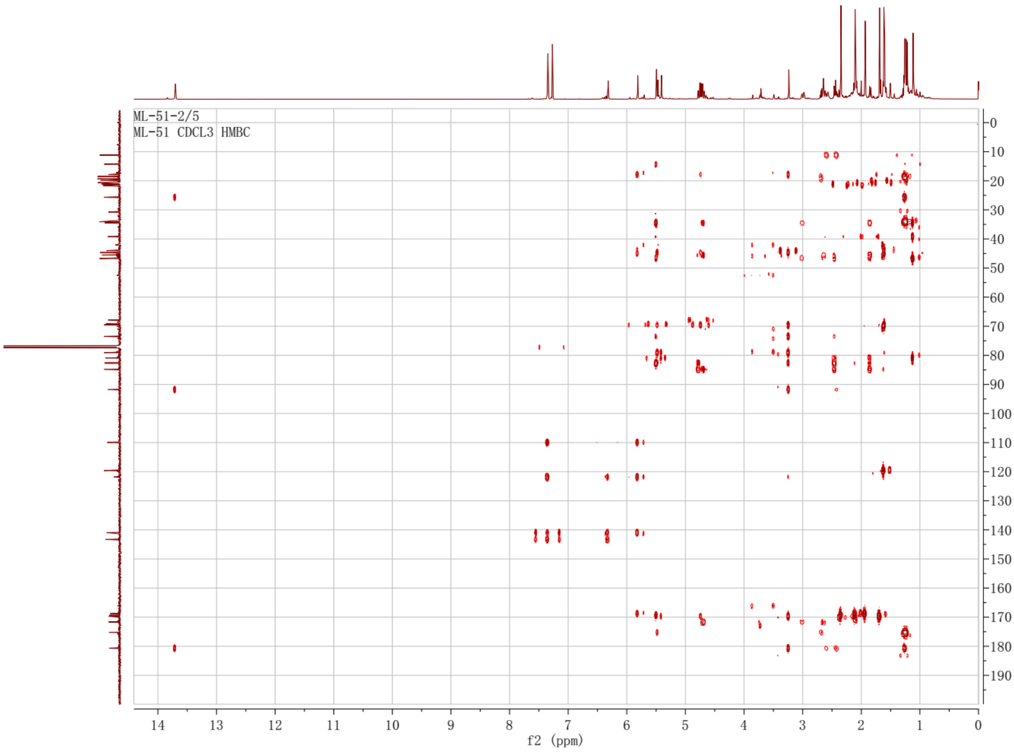

Figure S46. HMBC spectrum of **6**

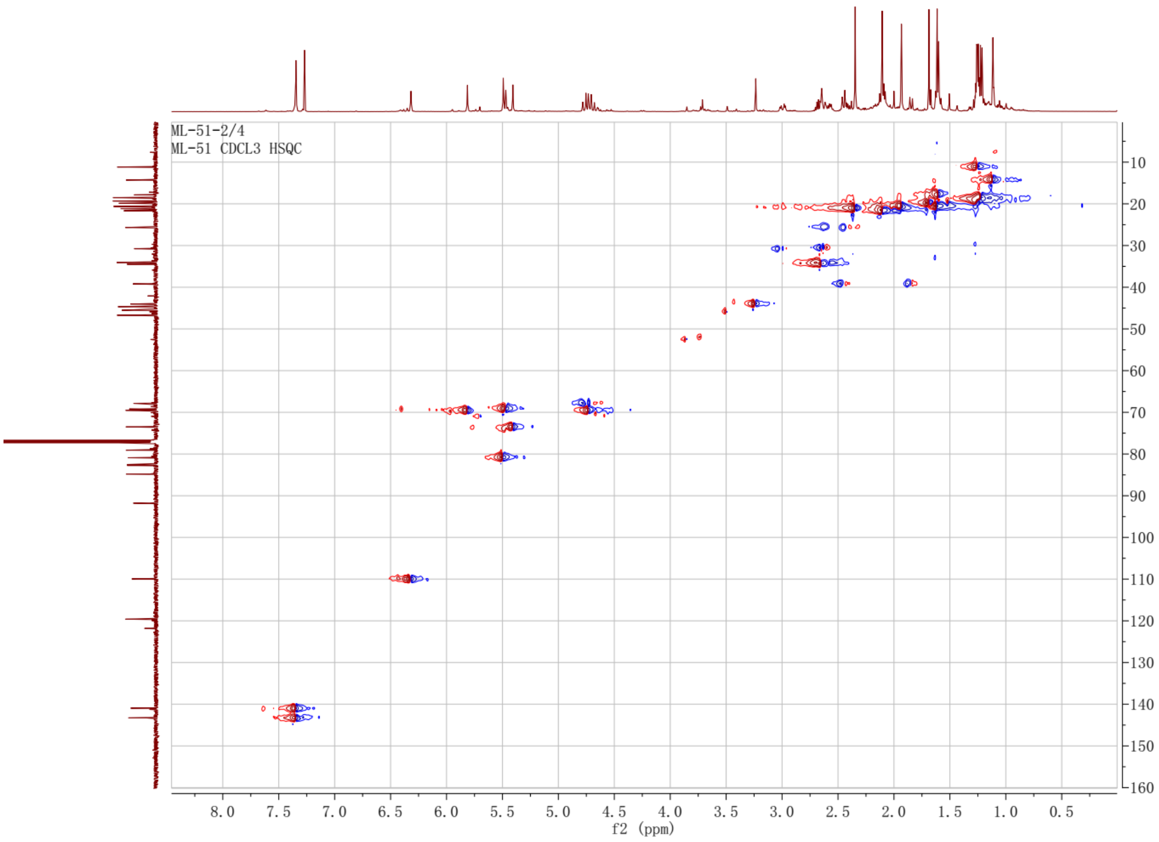

Figure S47. HSQC spectrum of **6**

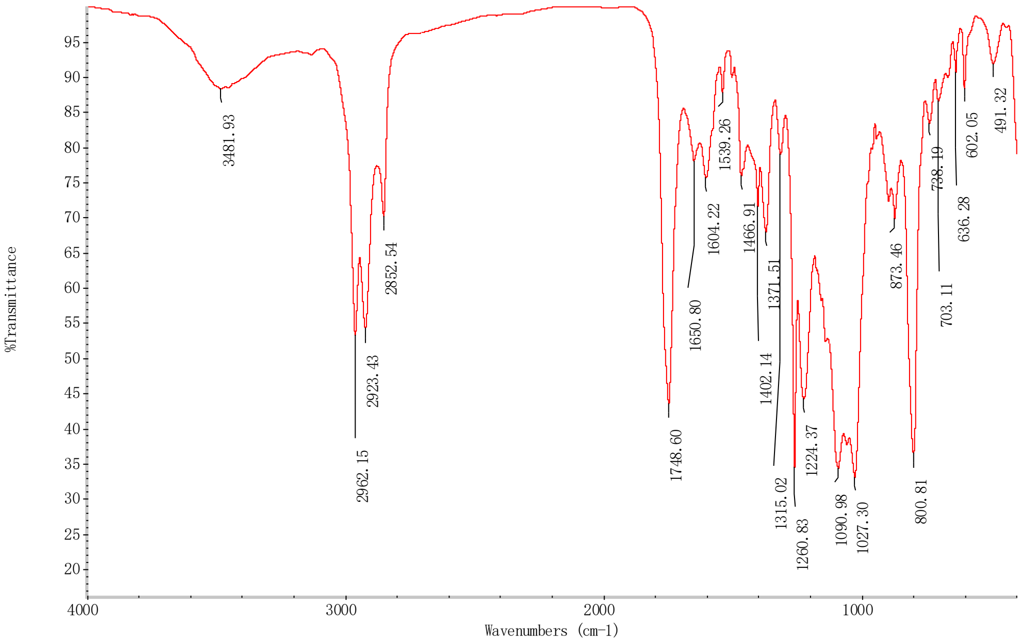

Figure S48. IR spectrum of **6**
